# Hypercapnia promotes microglial pyroptosis via inhibiting mitophagy in hypoxemic adult rats

**DOI:** 10.1111/cns.13435

**Published:** 2020-07-14

**Authors:** Hong‐guang Ding, Ya Li, Xu‐sheng Li, Xin‐qiang Liu, Kang‐rong Wang, Miao‐yun Wen, Wen‐qiang Jiang, Hong‐ke Zeng

**Affiliations:** ^1^ Department of Emergency and Critical Care Medicine Guangdong Provincial People’s Hospital Guangdong Academy of Medical Sciences Guangzhou China; ^2^ School of Medicine South China University of Technology Guangzhou China; ^3^ Southern Medical University Guangzhou China

**Keywords:** acute respiratory distress syndrome, hypercapnia, hypoxemia, mitophagy, pyroptosis

## Abstract

**Background:**

Hypoxemia is a typical symptom of acute respiratory distress syndrome. To avoid pulmonary morbidity, low tidal volume ventilation is often applied. The ventilation strategy will certainly cause hypercapnia. This study aimed to explore whether hypercapnia would promote microglial pyroptosis via inhibiting mitophagy in adult rats with hypoxemia.

**Methods:**

The cerebral oxygen extraction ratio (CERO_2_) and partial pressure of brain tissue oxygen (PbtO_2_) in a rat model of hypercapnia/hypoxemia were assessed. The reactive oxygen species (ROS) production and the expression of LC3‐II/I, p62, caspase‐1, gasdermin D‐N domains (GSDMD‐N), IL‐1β, and IL‐18 in microglial cells were detected.

**Results:**

Hypercapnia decreased the PbtO_2_ levels of the hypoxic rats, which was further evidenced by the increased levels of CERO_2_. Expression levels of LC3‐II were reduced, while p62 expression was increased by hypercapnia in hypoxic microglia. Hypercapnia increased the production of ROS and the expression of caspase‐1, GSDMD‐N, IL‐1β, and IL‐18 in hypoxia‐activated microglia. Scavenging ROS inhibited microglial pyroptosis and expression of IL‐1β and IL‐18.

**Conclusions:**

These results suggest that hypercapnia‐induced mitophagy inhibition may promote pyroptosis and enhance IL‐1β and IL‐18 release in hypoxia‐activated microglia.

## Graphical abstract

Hypercapnia‐induced mitophagy inhibition promotes pyroptosis and enhances IL‐1β and IL‐18 release in hypoxia‐activated microglia.

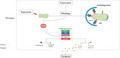

## 1 INTRODUCTION

Acute respiratory distress syndrome (ARDS) is a common critical disease in intensive care units (ICUs).^1^, ^2^ Persistent hypoxemia is a typical symptom of ARDS. Ventilations are always needed to correct hypoxemia.^3^, ^4^ To avoid or reduce pulmonary morbidity, low tidal volume ventilation is often applied.^5^, ^6^ The ventilation strategy will certainly result in hypercapnia, which is called “permissive hypercapnia”.^7^, ^8^ Our previous study found that hypercapnia could induce IL‐1β overproduction in hypoxia‐activated microglia.^9^ However, the underlying mechanism has remained unclear.

The NLRP3 inflammasome is widely expressed in microglia, which plays an important role in initiating the central immune and inflammatory response.^10^, ^11^, ^12^ To exert its functions, the NLRP3 inflammasome requires to be activated. The NLRP3 inflammasome can be triggered by an array of stimuli, including ATP, potassium efflux, heme, urate, and reactive oxygen species (ROS).^13^, ^14^, ^15^, ^16^, ^17^ Activation of the NLRP3 inflammasome can upregulate the expression of caspase‐1 and then leads to interleukin (IL)‐18 and IL‐1β maturation and pyroptosis activation. Pyroptosis is a proinflammatory cell death.^18^ Recent studies have reported that gasdermin D (GSDMD) is the executioner of pyroptosis.^19^ After being cleaved by caspase‐1, gasdermin D‐N domains (GSDMD‐N) can form a pore in the plasma membrane. IL‐1β and IL‐18 are released through the pores, thereby perpetuating cascade inflammatory response.^20^

Mitochondria‐derived ROS is the key stimuli to trigger the activation of NLRP3 inflammasome.^21^ When the levels of oxygen fall, hypoxia would damage mitochondria and induce ROS production.^22^, ^23^, ^24^ Mitophagy can eliminate damaged mitochondria, reduce ROS production, and alleviate NLRP3 inflammasome activation.^25^ It remains to be ascertained whether hypercapnia would activate the NLRP3 inflammasome through inhibiting mitophagy in hypoxia‐activated microglia.

In the present study, we hypothesized that hypercapnia may promote microglial pyroptosis via inhibiting mitophagy in hypoxemic adult rats. It was surmised that hypercapnia might exert its effect through inhibiting mitophagy, inducing ROS production, activating NLRP3 inflammasome, which ultimately would lead to IL‐18 and IL‐1β maturation and pyroptosis activation.

## 2 MATERIALS AND METHODS

### 2.1 Animals and experimental groups

The Experimental Animal Care and Use Committee at Jinan University approved all animal procedure protocols (No. 20 171 011 001), which conform to the Guide for the Care and Use of Laboratory Animals published by the US National Institutes of Health. All efforts were made to minimize the numbers of animals used and ensure minimal suffering. Male adult Sprague‐Dawley (SD) rats (n = 128) aged about 3 months and weighing approximately 220 to 250 g were randomly divided into five groups: sham‐operated group (abbreviated Sham group), Hypercapnia group, Hypoxemia group, Hypercapnia + Hypoxemia group (abbreviated HH group), Hypercapnia + Hypoxemia +N‐Acetyl‐L‐cysteine group (abbreviated HH + NAC group).

### 2.2 The rat model of hypercapnia/hypoxemia

All rats were fasted with access to water overnight before the experiments. The rat model of hypercapnia/hypoxemia was established as described in our previous study.^9^ Briefly, the rats were anesthetized with pentobarbital sodium (30 mg/kg) followed by mechanical ventilation. The tidal volume (9 mL/kg body weight), respiratory rate (45 breaths/min), and inspiratory‐to‐expiratory ratio (1:1) were fixed. Sham group was exposed to the air. Hypercapnia group was exposed to 5% CO_2_ to maintain the potential of hydrogen (pH) of arterial blood at 7.20–7.25. Hypoxemia group was exposed to 16% O_2_ to maintain partial pressure of arterial blood oxygen (PaO_2_) at 55–60 mmHg. HH group was exposed to 16% O_2_ mixing with 5% CO_2_ to maintain PaO_2_ at 55–60 mmHg and pH at 7.20–7.25. Rats in the HH + NAC group were treated with an intraperitoneal injection of 150 mg/kg NAC (MedChemExpress, Monmouth, NJ, USA; cat. no. HY‐B0215) for 30 minutes before being exposed to 5% CO_2_ and 16% O_2_. The rats, which were used for Western blotting analysis, immunofluorescence staining and ROS evaluation, were not performed invasive manipulation except ventilation. The rats were executed immediately after hypercapnia/hypoxemia treatment.

### 2.3 Measurement of cerebral oxygen extraction ratio

The right femoral artery and the right jugular vein were cannulated upstream. The blood samples were collected at 3 hours after ventilation. The hemoglobin concentration (Hb), saturation of arterial blood oxygen (SaO_2_), PaO_2_, saturation of jugular venous blood oxygen (SjVO_2_), and pressure of jugular venous blood oxygen (PjVO_2_) were measured using a blood gas/electrolyte analyzer (Model 5700; San Diego, CA, USA). The content of arterial blood oxygen (CaO_2_), content of jugular venous blood oxygen (CjVO_2_), and cerebral oxygen extraction ratio (CERO_2_) were calculated using the following formulas:

$${\text{CaO}}_{2}=\text{Hb}\times 1\text{.36}\times {\text{SaO}}_{2}+0\text{.0031}\times {\text{PaO}}_{2}$$

$${\text{CjVO}}_{2}=\text{Hb}\times 1\text{.36}\times {\text{SjVO}}_{2}+0\text{.0031}\times {\text{PjVO}}_{2}$$

$${\text{CERO}}_{2}{\;\text{= (CaO}}_{2}{\;‐\;\text{CjVO}}_{2}{\text{)/CaO}}_{2}$$

### 2.4 Measurement of the partial pressure of brain tissue oxygen (PbtO_2_)

The levels of PbtO_2_ were measured at 0.5, 1, 1.5, 2, 2.5, and 3 hours after ventilation. To evaluate the PbtO_2_, a midline incision over vertex was performed after anesthesia. After this, a hole was drilled caudal to the coronal suture, 4 mm from the midline. The dura was punctured, and a microsensor for PbtO_2_ was inserted into brain tissue.^26^ A monitor (Integra CAMO2, Integra LifeSciences Limited, County Offaly, Ireland) was used to measure the PbtO_2_.

### 2.5 ROS evaluation of brain tissue

The ROS of brain tissue was evaluated using a ROS ELISA kit (Dogesce, Beijing, China; cat. no. DG21175D) at 3 hours after ventilation following the manufacturers’ instructions. Briefly, samples and standards (50 μL/well) were added to the plate wells coated by antibodies labeled with HRP, which were used to capture ROS. The plate was incubated for 1 hour at 37°C. After washing completely, substrate A (50 μL/well) and substrate B (50 μL/well) were added to incubate the plate in a dark place for 15 minutes at 37°C. Then, the stop buffer was added, and the optical density (OD) was measured spectrophotometrically at a wavelength of 450 nm. The concentrations of ROS in the samples were then determined by comparing the optical density of the samples to the standard curve.

### 2.6 BV‐2 microglial cell cultures and treatment

BV‐2 microglial cells were purchased form CHI Scientific (cat. no. 7‐1502) and were cultured and treated as described in our previous study.^9^ Briefly, the cells were cultured in DMEM high glucose (Invitrogen Life Technologies Corporation, Carlsbad, CA, USA; cat. no. 8 117 121) supplemented with 10% FBS (Invitrogen Life Technologies Corporation; Carlsbad, CA, USA; cat. no. 42F0374K) in a humidified incubator with 5% CO_2_/95% air. The microglial cells were randomly divided into five groups: Control group, high concentration of carbon dioxide group (abbreviated HC group), Hypoxia group, Hypoxia + HC group, and Hypoxia + HC +NAC group. Control group was exposed to 5% CO_2_ + 20% O_2_. HC group was exposed to 15% CO_2_ + 20% O_2_ to maintain the pH of the supernatant at 7.20‐7.25. Hypoxia group was exposed to 5% CO_2_ + 0.2% O_2_ to maintain partial pressure of oxygen (PO_2_) of the supernatant at 55‐60 mmHg. Hypoxia + HC group was exposed to 15% CO_2_ + 0.2% O_2_ to maintain PO_2_ at 55‐60 mmHg and pH at 7.20‐7.25. The cells in the Hypoxia + HC + NAC group were treated with 2 mmol/L NAC (MedChemExpress, Monmouth, NJ, USA; cat. no. HY‐B0215) for 30 minutes before being exposed to 15% CO_2_ + 0.2% O_2_.

### 2.7 Oxygen consumption rate evaluation of BV‐2 microglial cells

**FIGURE 1:**
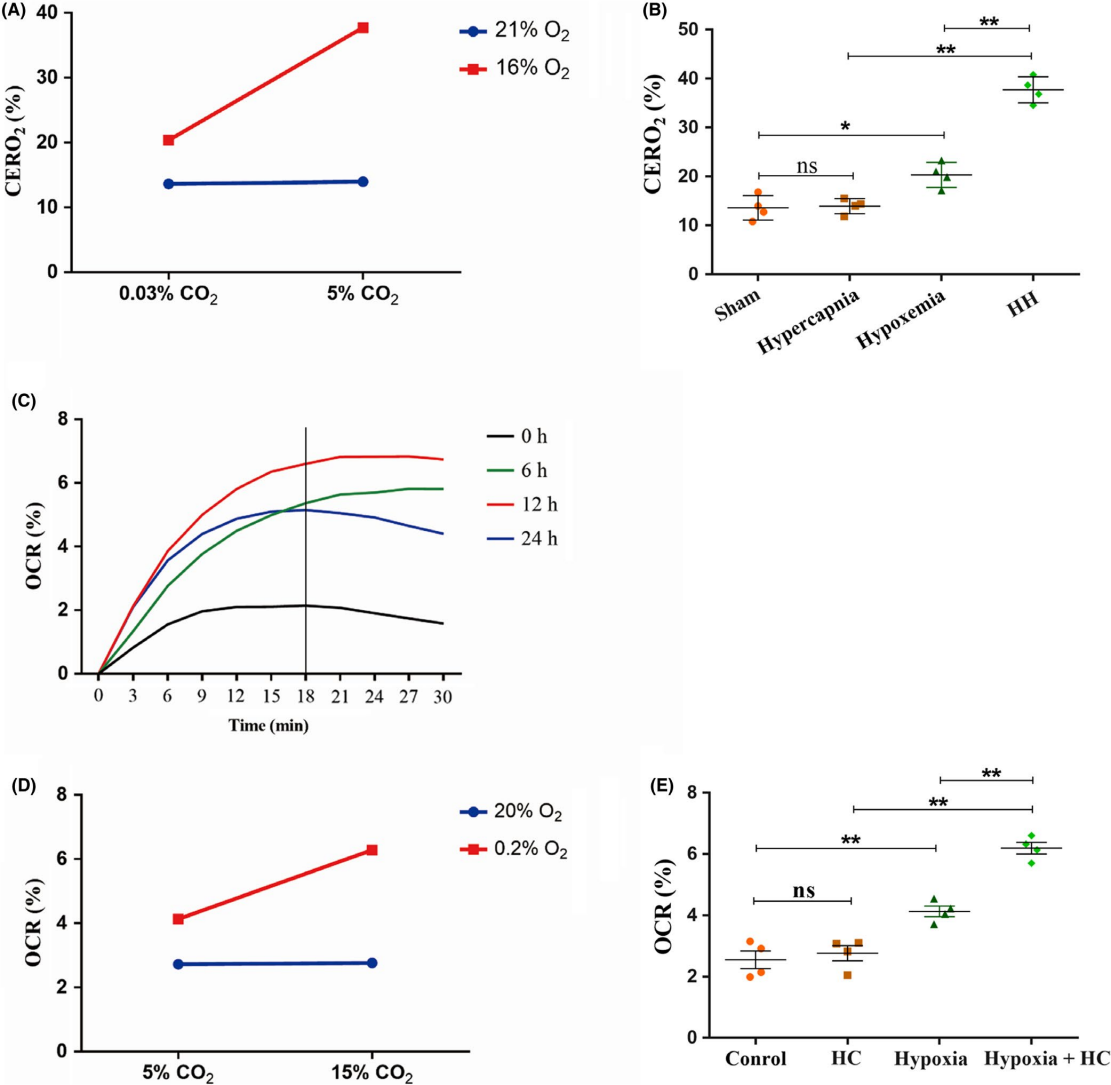
Hypercapnia increased CERO_2_ and OCR in vivo and in vitro (n = 4). (A) There is an interaction effect between hypoxia treatment and hypercapnia treatment (*P* < .01). (B) Simple effects analyses show an increased CERO_2_ in Hypoxemia group (*P < .05), but not in Hypercapnia group (ns* P* > .05) compared with Sham group. HH group has the highest CERO_2_ levels as compared with Hypoxemia group (**P < .01) and Hypercapnia group (**P < .01). (C) The OCR levels are the highest in 12 h group as compared with other groups (12 h vs 0 h: *P* < .01; 12 h vs 6 h: *P* > .05; 12 h vs 24 h: *P* < .01). The OCR levels are increased time‐dependently and achieved a stationary phase at 18 min. So the microglia cells were treated for 12 hours, and the OCR was examined at 18 min after the treatment, as shown in graph (D) and (E). (D) There is an interaction effect between 0.2% O_2_ treatment and 15% CO_2_ treatment (*P* < .01). (E) Simple effects analyses show increased levels of OCR in Hypoxia group (**P < .01), but not in HC group (ns *P* > .05) compared with Control group. Hypoxia + HC group has the highest levels of OCR as compared with Hypoxia group (**P < .01) and HC group (**P < .01). ns, nonsignificant; Sham group, sham‐operated group; HH group, hypercapnia + hypoxemia group; HC group, high concentration of carbon dioxide group; CERO_2_, cerebral oxygen extraction rate; OCR, oxygen consumption rate. The concentrations of O_2_ and CO_2_ in the air are 21% and 0.03%

The Oxygen consumption rate (OCR) was evaluated using a cellulate OCR Assay Kit (cat. no. BB‐48211; BestBio, Shanghai, China) after treatment with 0.2% O_2_ and 15% CO_2_ for 0 hour, 6 hours, 12 hours, and 24 hours. The intervention time and testing time were chosen, when the levels of OCR peaked (In this study, 12 hours was chosen as the intervention time, and 18 minutes was chosen as the testing time) (Figure 1C). The OCR was measured following the manufacturers’ instructions. Briefly, BV‐2 microglial cells were seeded in 96‐well plates (5000 cells/well) and cultured in DMEM high glucose supplemented with 10% FBS. When the cells spread to 80% of the bottom of the well, they were treated with different concentrations of CO_2_ and O_2_. After the treatment, the medium was changed to DMEM high glucose without FBS, and then, fluorescent probes (10 μL/well) were added sequentially. Finally, an oxygen mounting medium was added (2 drops/well). The OCR levels were examined every three minutes until half an hour with a fluorescent microplate reader (Model 9260; IL‐COR^®^ inc, Lincoln, NE, USA). The excited and emitted wavelengths were 485/20 nm and 590/35 nm, respectively.

### 2.8 ROS measurement in microglia

The ROS production in BV‐2 microglial cells was evaluated using a ROS assay kit (cat. no. BB‐4705‐2; BestBio, Shanghai, China) following the manufacturers’ instructions. Briefly, DCFH‐DA was diluted with DMEM high glucose without FBS (1:1500). The coverslips with adherent BV‐2 microglial cells were cultured with DMEM high glucose supplemented with 10% FBS. After the treatment, the medium was changed to diluted DCFH‐DA (2 mL/well). Then, the plates were incubated for 20 minutes at 37°C, 5% CO_2_. The coverslips were washed with DMEM high glucose without FBS. Finally, the coverslips were mounted by a fluorescent mounting medium and detected using a fluorescence microscope (Olympus DP73 Microscope, Olympus, Tokyo, Japan).

### 2.9 Western blotting analysis

Total proteins from the hippocampus tissue and BV‐2 microglial cells (n = 4 for each group) were extracted using a Total Protein Extraction Kit (BestBio, Shanghai, China; cat. no. BB‐3101‐100T). Protein concentrations were determined using a BCA Protein Assay Kit (Invitrogen Life Technologies Corporation, Carlsbad, CA, USA; cat. no. 23 227). Equal amounts of protein (40 μg) from each sample were separated in a 15% SDS‐PAGE gel and transferred to PVDF membranes, which were blocked with 5% nonfat milk for 1 hours at room temperature. After this, the following primary antibodies were added to incubate the membranes overnight at 4°C: LC3B antibody (1:1000, Abcam, Cambridge, MA, USA; cat. no. ab48394), SQSTM1/p62 (1:1000, Abcam, Cambridge, MA, USA; cat. no. ab56416), Gasdermin D (1:1000, Cell Signaling Technology; cat. no. 93 709), caspase‐1 (1:1000, Abcam, Cambridge, MA, USA; cat. no. ab1872), IL‐18 (1:1000, Abcam, Cambridge, MA, USA; Cat. No. ab207323), and IL‐1β (1:1000, Abcam, Cambridge, MA, USA; Cat. No. ab9722). The membranes were washed on the following day, and the HRP‐labeled goat antirabbit antibody (1:3000; Cell Signaling Technology; cat. no. 7074S) was added to incubate the membranes for 2 hours at 4°C. The immunoblots were visualized using a chemiluminescence kit (Bioworld Technology, St. Louis Park, MN, USA; cat. no. AC36131) and detected by an imaging densitometer (ImageQuant LAS 500, GE Healthcare Bio‐Sciences AB, Uppsala, Sweden). The relative density was quantified using FluorChem 8900 software (version 4.0.1, Alpha Innotech Corporation, San Leandro CA, USA). β‐actin was used as the control.

### 2.10 Double immunofluorence labeling

In vivo, the rats were anesthetized with pentobarbital sodium and transcardially perfused with normal saline and 4% paraformaldehyde at 3 hours after ventilation. The brains were harvested and postfixed in 4% paraformaldehyde. This tissue was then dehydrated in graded sucrose and cut into sections of 10 μm thickness. The sections were blocked in 5% normal donkey serum for 0.5 hours at room temperature. In vitro, the coverslips with adherent BV‐2 microglial cells were fixed with 4% paraformaldehyde at 24 hours after treatment. The coverslips were blocked in 5% normal donkey serum for 0.5 hours at room temperature sequentially.

After that, the following primary antibodies were added to incubate the sections/coverslips overnight at 4°C: caspase‐1 (1:100; Abcam, Cambridge, MA, USA; cat. no. ab1872), IL‐1β (1:100; Abcam, Cambridge, MA, USA; cat. no. ab9722), IL‐18 (1:100, Abcam, Cambridge, MA, USA; Cat. No. ab207323), and Iba1 (1:100; Abcam, Cambridge, MA, USA; cat. no. ab15690). The sections/coverslips were washed on the following day, and the secondary antibodies Alexa Fluor^®^ 549 Goat antirabbit IgG (H + L) (1:100; Invitrogen Life Technologies, Carlsbad, CA, USA; cat. no. ATRJN1301) and Alexa Fluor^®^ 488 Goat antimouse IgG (1:100; Invitrogen Life Technologies, Carlsbad, CA, USA; cat. no. ATRMR2301) were added to incubate the sections/coverslips for 1 h at room temperature. Finally, the sections/coverslips were mounted by the fluorescent mounting medium with DAPI (Sigma, St. Louis, MO, USA; cat. no. SLBW4468) and detected using a fluorescence microscope (Olympus DP73 Microscope, Olympus, Tokyo, Japan).

### 2.11 Statistical analysis

The statistical analysis was performed by the SPSS19.0 statistical (IBM, New York, USA). All values are expressed as mean ± standard error of mean (SEM). Shapiro‐Wilk test was used to assess the data distribution. All data were normally distributed in the present study. Repeated measures one‐way analysis of variance (ANOVA) was used to analyze the repeated measurement data. Factorial ANOVA was for the interaction effects. When an interaction was examined, simple effects analyses were evaluated. Differences were considered statistically significant if the *P‐*value < .05.

## 3 RESULTS

### 3.1 Hypercapnia increased cerebral oxygen extraction rate in hypoxemic rats

Significant interaction effects were observed between hypercapnia treatment and hypoxia treatment (*P* < .01) (Figure 1A). Simple effects analyses found an increased CERO_2_ in Hypoxemia group (*P* < .05), but not in Hypercapnia group (*P* > .05) compared with Sham group. HH group had the highest CERO_2_ levels as compared with Hypoxemia group (*P* < .01) and Hypercapnia group (*P* < .01) (Figure 1B).

### 3.2 15% CO_2_ increased the oxygen consumption rate (OCR) in hypoxic BV‐2 microglial cells

The OCR levels were the highest in 12 hours group as compared with other groups (12 hours vs 0 hour: *P* < .01; 12 hours vs 6 hours: *P* > .05; 12 hours vs 24 hours: *P* < .01). Besides, the OCR levels were increased time‐dependently and achieved a stationary phase at 18 minutes (Figure 1C). Because of this, microglia were treated for 12 hours, and the OCR was examined at 18 minutes after the treatment (Figure 1D, E). Significant interaction effects were observed between 0.2% O_2_ treatment and 15% CO_2_ treatment (*P* < .01; Figure 1D). Simple effects analyses found increased levels of OCR in Hypoxia group (*P* < .01), but not in HC group (*P* > .05) compared with Control group. Hypoxia + HC group had the highest levels of OCR as compared with Hypoxia group (*P* < .01) and HC group (*P* < .01) (Figure 1E).

### 3.3 Hypercapnia decreased partial pressure of brain tissue oxygen (PbtO_2_) in hypoxemic rats

**FIGURE 2:**
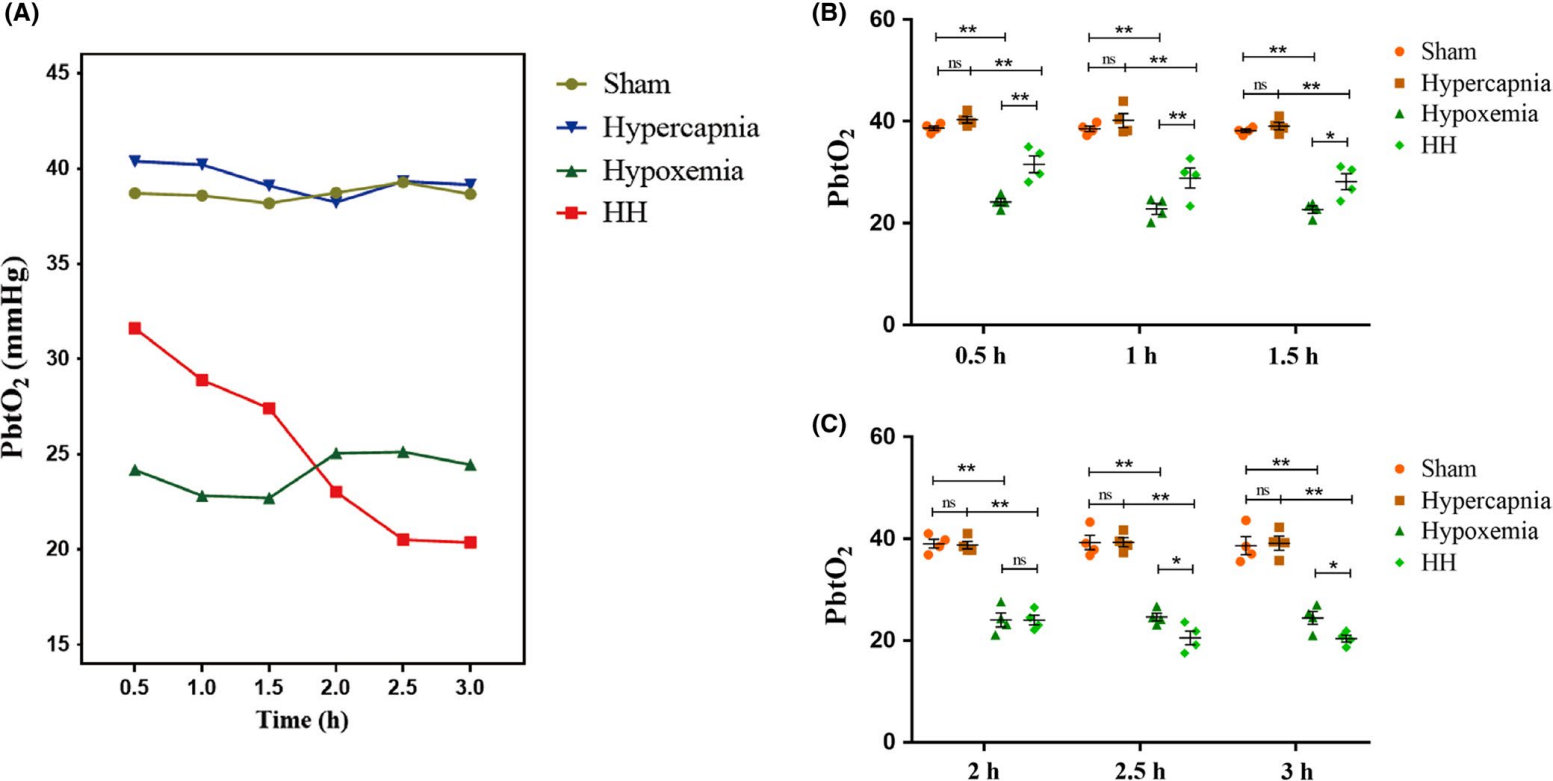
Hypercapnia decreased PbtO_2_ in hypoxemic rats (n = 4). Graphs (A) illustrate the PbtO_2_ at 0.5, 1, 1.5, 2, 2.5, and 3 h after ventilation in the rats. (B, C) Compared with the Sham group, PbtO_2_ levels are decreased in Hypoxemia group (0.5 h‐3 h: *P* < .01), but not in Hypercapnia group (0.5 h‐3 h: *P* > .05). HH group has higher PbtO_2_ levels as compared with Hypoxemia group in the first 1.5 h (0.5 h: *P* < .01; 1 h: *P* < .01; 1.5 h: *P* < .05). On the contrary, HH group has lower PbtO_2_ levels as compared with Hypoxemia group after 2 h (2.5 h: *P* < .05; 3 h: *P* < .05). Sham group, sham‐operated group; HH group, hypercapnia + hypoxemia group; PbtO_2_, partial pressure of brain tissue oxygen

HH group had higher PbtO_2_ levels as compared with Hypoxemia group in the first 1.5 hours (0.5 hours: *P* < .01; 1 hour: *P* < .01; 1.5 hours: *P* < .05) (Figure 2A, B). On the contrary, HH group had lower PbtO_2_ levels as compared with Hypoxemia group after 2 hours (2.5 hours: *P* < .05; 3 hours: *P* < .05) (Figure 2A, C).

### 3.4 Hypercapnia inhibited mitophagy in microglia in the hypoxic hippocampus

**FIGURE 3:**
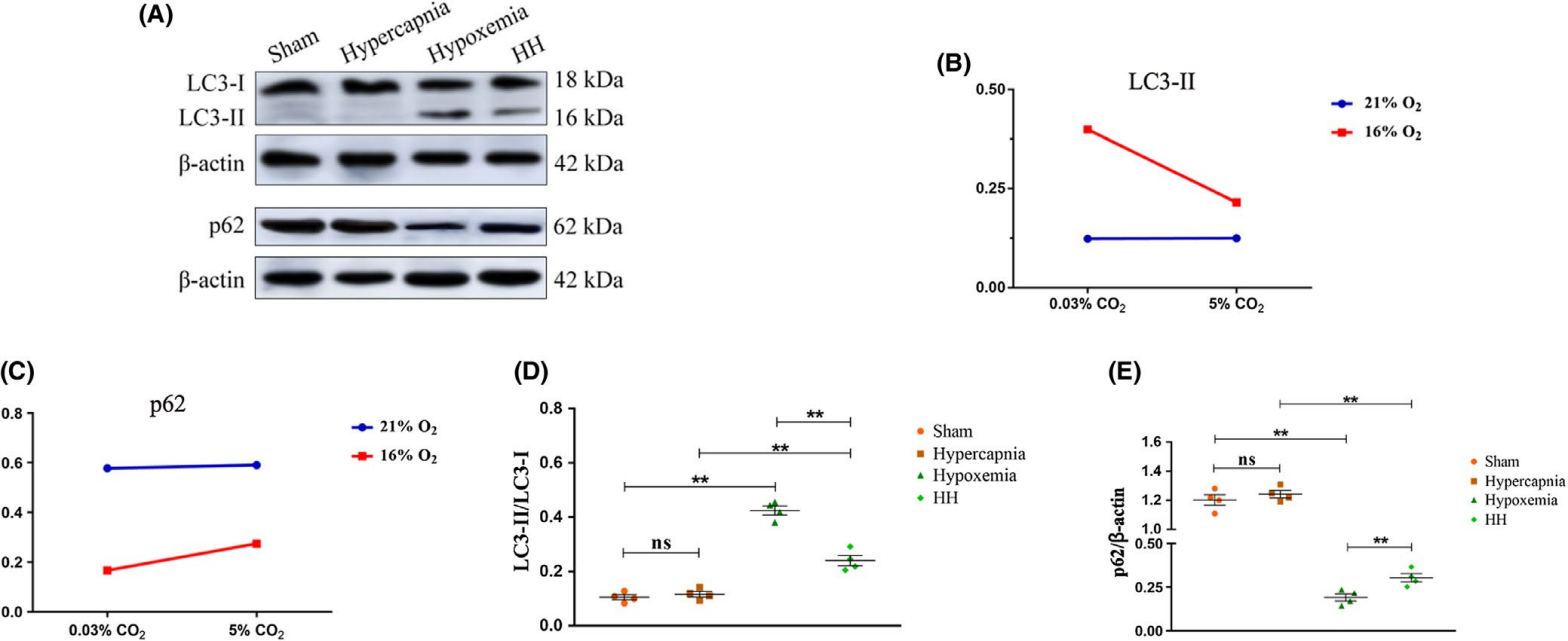
Hypercapnia inhibited mitophagy in microglia in the hypoxic hippocampus (n = 4). (A) Immunoreactive bands of LC3‐I (18 kDa), LC3‐II (16 kDa), p62 (62 kDa), and β‐actin (42 kDa). (B, C) There is an interaction effect between hypoxia treatment and hypercapnia treatment (LC3‐II: *P* < .01, p62: *P* < .01). (D) Simple effects analyses show increased protein expression levels of LC3‐II in Hypoxemia group compared with Sham group (**P < .01). HH group shows lower expression levels of LC3‐II in comparison with Hypoxemia group (**P < .01). (E) In contrast, a significant decrease in p62 expression is observed in Hypoxemia group compared with Sham group (**P < .01). The levels of p62 expression in HH group are higher compared with Hypoxemia group (**P < .01). ns, nonsignificant; Sham group, sham‐operated group; HH group, hypercapnia + hypoxemia group. The concentrations of O_2_ and CO_2_ in the air are 21% and 0.03%

Significant interaction effects were observed between hypercapnia treatment and hypoxia treatment (LC3‐II: *P* < .01, p62: *P* < .01) (Figure 3B, C). Simple effects analyses found increased protein expression levels of LC3‐II in Hypoxemia group compared with Sham group (*P* < .01). HH group showed lower expression levels of LC3‐II in comparison with Hypoxemia group (*P* < .01) (Figure 3A, D). In contrast, a significant decrease in p62 expression was observed in Hypoxemia group compared with Sham group (*P* < .01). The levels of p62 expression in HH group were higher compared with Hypoxemia group (*P* < .01) (Figure 3E). The full Western blots of the above each group were shown in Figure S7.

### 3.5 15% CO_2_ inhibited mitophagy in hypoxia‐activated BV‐2 microglia

**FIGURE 4:**
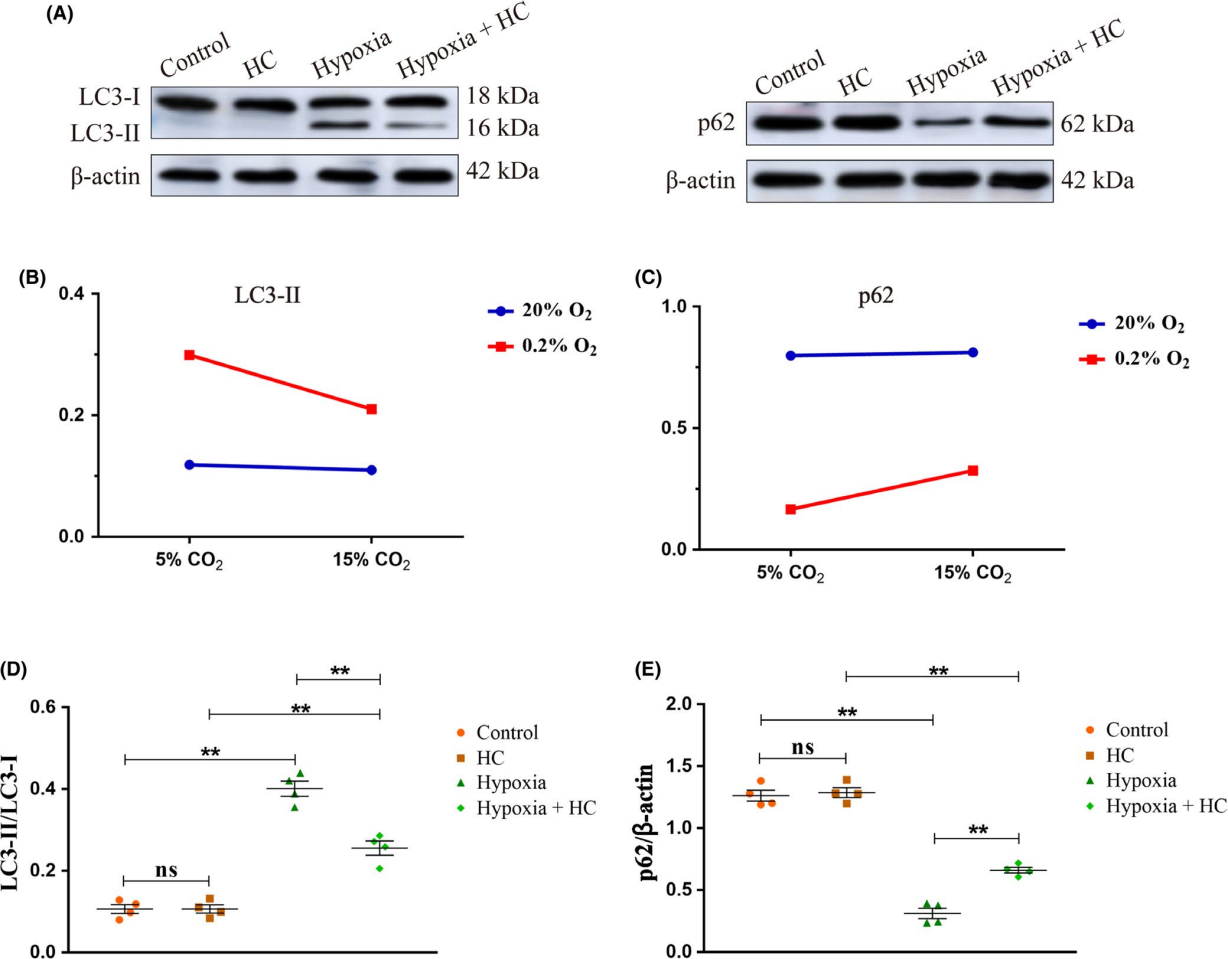
15% CO_2_ inhibited mitophagy in hypoxia‐activated BV‐2 microglia (n = 4). (A) Immunoreactive bands of LC3‐I (18 kDa), LC3‐II (16 kDa), p62 (62 kDa), and β‐actin (42 kDa). (B, C) There is an interaction effect between 0.2% O_2_ treatment and 15% CO_2_ treatment (LC3‐II: *P* < .01, p62: *P* < .01). (D) Simple effects analyses show increased protein expression levels of LC3‐II in Hypoxia group compared with Control group (*P* < .01). Hypoxia + HC group shows lower expression levels of LC3‐II in comparison with Hypoxia group (*P* < .01). (E) In contrast, a significant decrease in p62 expression is observed in Hypoxia group compared with Control group (*P* < .01). The levels of p62 expression in Hypoxia + HC group are higher compared with Hypoxia group (*P* < .01). ns, nonsignificant; HC group, high concentration of carbon dioxide group

Significant interaction effects were observed between 0.2% O_2_ treatment and 15% CO_2_ treatment (LC3‐II: *P* < .01, p62: *P* < .01) (Figure 4B, C). Simple effects analyses found increased protein expression levels of LC3‐II in Hypoxia group (*P* < .01) compared with Control group. Hypoxia + HC group showed lower expression levels of LC3‐II in comparison with Hypoxia group (*P* < .01) (Figure 4A, D). In contrast, a significant decrease in p62 expression was observed in Hypoxia group compared with Control group (*P* < .01). The levels of p62 expression in Hypoxia + HC group were higher compared with Hypoxia group (*P* < .01) (Figure 4E). The full Western blots of the above each group were shown in Figure S8.

### 3.6 Hypercapnia induced overproduction of ROS in the hypoxemic hippocampus

**FIGURE 5:**
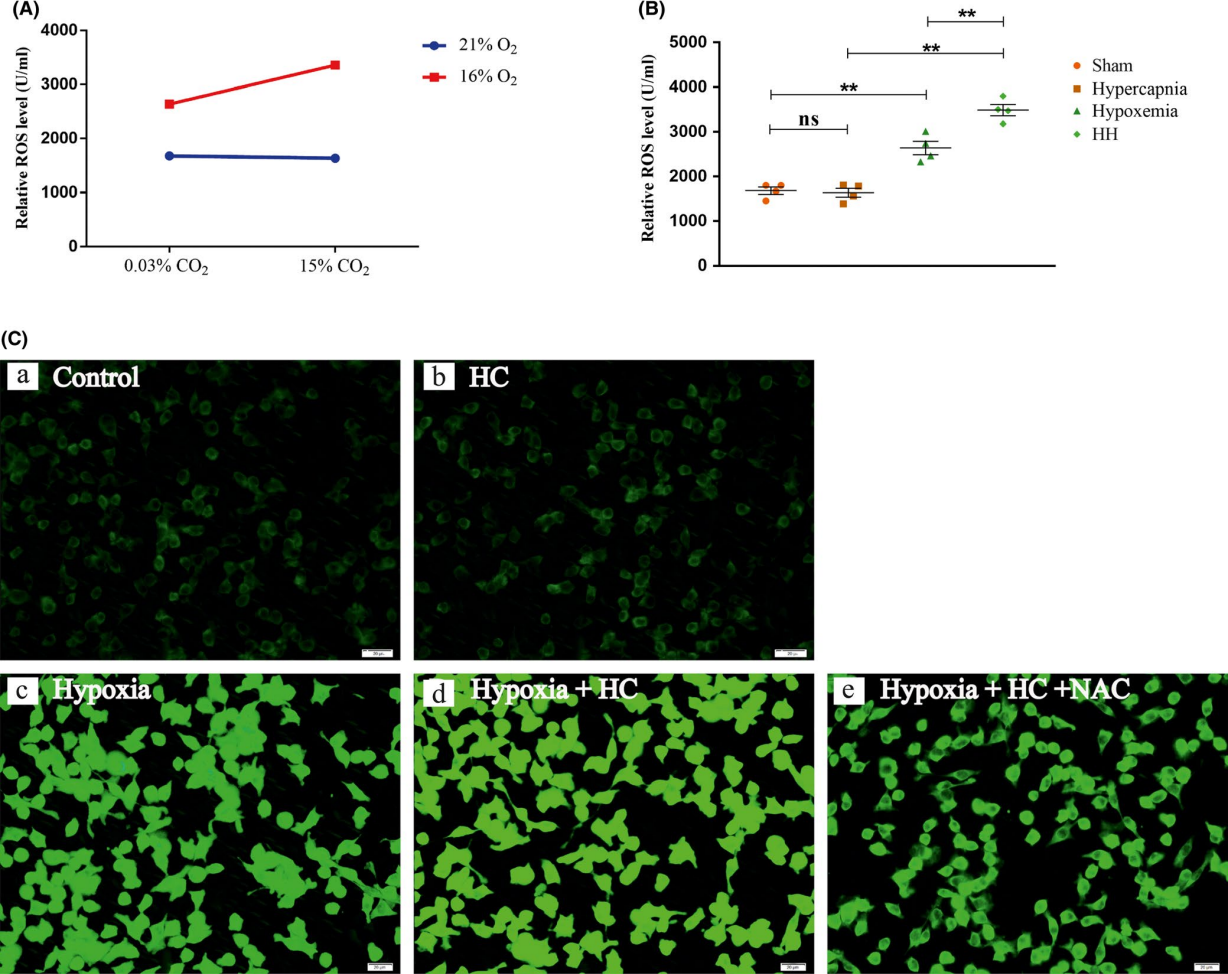
Hypercapnia induced overproduction of ROS in the hypoxemic hippocampus and BV2 cells (n = 4). (A) There is an interaction effect between hypoxia treatment and hypercapnia treatment (*P* < .05). (B) Simple effects analyses show increased levels of ROS in Hypoxemia group (**P < .01), but not in Hypercapnia group (ns *P* > .05) compared with Sham group. HH group has the highest ROS levels as compared with Hypoxemia group (**P < .01) and Hypercapnia group (**P < .01). (C) Immunofluorescence images showing the production of ROS (a‐e, green). Enhanced ROS immunofluorescence is evident in Hypoxia group, but not in HC group compared with Control group. Hypoxia + HC group exhibits the most intense ROS fluorescence as compared with Hypoxia group and HC group. ROS fluorescence is noticeably reduced with NAC pretreatment (2 mmol/L) in BV‐2 microglial cells. Scale bars: 20 μm. ns, nonsignificant; Sham group, sham‐operated group; HH group, hypercapnia + hypoxemia group; ROS, reactive oxygen species; HC group, high concentration of carbon dioxide group. The concentrations of O_2_ and CO_2_ in the air are 21% and 0.03%

Significant interaction effects were observed between hypercapnia treatment and hypoxia treatment (*P* < .05) (Figure 5A). Simple effects analyses found increased levels of ROS in Hypoxemia group (*P* < .01), but not in Hypercapnia group (*P* > .05) compared with Sham group. HH group had the highest ROS levels as compared with Hypoxemia group (*P* < .01) and Hypercapnia group (*P* < .01) (Figure 5B).

### 3.7 15% CO_2_ induced overproduction of ROS in hypoxic BV‐2 microglial cells

Enhanced ROS immunofluorescence was observed in Hypoxia group, but not in HC group compared with Control group. Hypoxia + HC group had the strongest ROS fluorescence as compared with Hypoxia group and HC group. ROS fluorescence was obviously reduced with NAC pretreatment (2 mmol/L) in BV‐2 microglial cells (Figure 5C).

### 3.8 Hypercapnia enhanced NLRP3 inflammasome activation via inducing ROS overproduction in microglia in the hypoxic hippocampus

**FIGURE 6:**
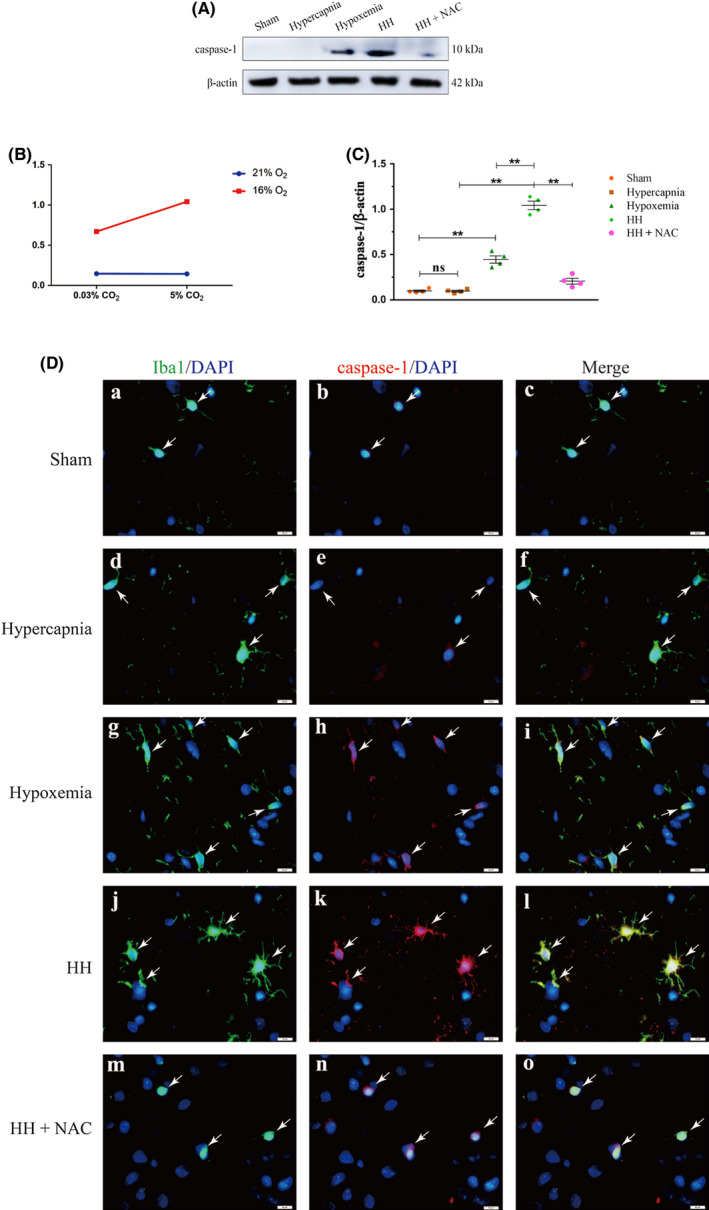
Hypercapnia enhanced NLRP3 inflammasome activation via inducing ROS overproduction in microglia of the hypoxic hippocampus (n = 4). (A) Immunoreactive bands of caspase‐1 (10 kDa) and β‐actin (42 kDa). (B) There is an interaction effect between hypoxia treatment and hypercapnia treatment (*P* < .01). (C) Simple effects analyses show increased protein expression levels of caspase‐1 in Hypoxemia group (**P < .01), but not in Hypercapnia group (ns *P* > .05) compared with Sham group. HH group has the highest expression levels of caspase‐1 as compared with Hypoxemia group (**P < .01) and Hypercapnia group (**P < .01). The protein expression of caspase‐1 is significantly suppressed with NAC pretreatment (150 mg/kg) in rats (**P < .01). (D) Immunofluorescence images showing the expression of Iba1^+^ microglia (a, d, g, j, m, green), caspase‐1 (b, e, h, k, n, red), and the colocalization of caspase‐1 and microglia (x, f, i, l, o). Enhanced caspase‐1 immunofluorescence is evident in Hypoxemia group, but not in Hypercapnia group compared with Sham group. HH group emits the strongest caspase‐1 fluorescence as compared with Hypoxemia group and Hypercapnia group. Caspase‐1 fluorescence is markedly attenuated in rats given NAC pretreatment. Scale bars: 10 μm. ns, nonsignificant; Sham group, sham‐operated group; HH group, hypercapnia + hypoxemia group. The concentrations of O_2_ and CO_2_ in the air are 21% and 0.03%

Significant interaction effects were observed between hypercapnia treatment and hypoxia treatment (*P* < .01) (Figure 6B). Simple effects analyses found increased protein expression levels of caspase‐1 in Hypoxemia group (*P* < .01), but not in Hypercapnia group (*P* > .05) compared with Sham group. HH group had the highest expression levels of caspase‐1 as compared with Hypoxemia group (*P* < .01) and Hypercapnia group (*P* < .01) (Figure 6A, C). Additionally, the protein expression of caspase‐1 was significantly suppressed with NAC pretreatment (150 mg/kg) in rats (*P* < .01) (Figure 6C). The full Western blots of the above each group were shown in Figure S9. Double immunofluorescence was used to examine caspase‐1 expression in microglia of the hippocampus (Figure 6D). Enhanced caspase‐1 immunofluorescence was observed in Hypoxemia group, but not in Hypercapnia group compared with Sham group. HH group had the most intense caspase‐1 fluorescence when compared with Hypoxemia group and Hypercapnia group. In rats given NAC treatment, caspase‐1 fluorescence was noticeably attenuated (Figure 6D).

### 3.9 15% CO_2_ enhanced NLRP3 inflammasome activation via inducing ROS overproduction in hypoxic BV‐2 microglial cells

Significant interaction effects were observed between 0.2% O_2_ treatment and 15% CO_2_ treatment (*P* < .01) (Figure S1B). Simple effects analyses found increased protein expression levels of caspase‐1 in Hypoxia group (*P* < .01), but not in HC group (*P* > .05) compared with Control group. Hypoxia + HC group had the highest expression levels of caspase‐1 as compared with Hypoxia group (*P* < .01) and HC group (*P* < .01) (Figure S1A, C). Additionally, the protein expression of caspase‐1 was significantly suppressed with NAC pretreatment (2 mmol/L) in BV‐2 microglial cells (*P* < .01) (Figure S1C). The full Western blots of the above each group were shown in Figure S10. Double immunofluorescence was used to examine caspase‐1 expression in BV‐2 microglial cells (Figure S1D). Enhanced caspase‐1 immunofluorescence was observed in Hypoxia group, but not in HC group compared with Control group. Hypoxia + HC group had the strongest caspase‐1 fluorescence as compared with Hypoxia group and HC group. Of note, caspase‐1 fluorescence was evidently reduced with NAC pretreatment (2 mmol/L) in BV‐2 microglial cells (Figure S1D).

### 3.10 Hypercapnia increased GSDMD‐N expression via inducing ROS overproduction in microglia in the hypoxic hippocampus

Significant interaction effects were observed between hypercapnia treatment and hypoxia treatment (*P* < .01) (Figure S2B). Simple effects analyses found increased protein expression levels of GSDMD‐N in Hypoxemia group (*P* < .05), but not in Hypercapnia group (*P* > .05) compared with Sham group. HH group showed the highest expression levels of GSDMD‐N in comparison with Hypoxemia group (*P* < .01) and Hypercapnia group (*P* < .01). Additionally, the protein expression of GSDMD‐N was significantly suppressed with NAC pretreatment (150 mg/kg) in rats (*P* < .01) (Figure S2A, C). The full Western blots of the above each group were shown in Figure S11.

### 3.11 15% CO_2_ increased GSDMD‐N expression via inducing ROS overproduction in hypoxic BV‐2 microglial cells

Significant interaction effects were observed between 0.2% O_2_ treatment and 15% CO_2_ treatment (*P* < .01) (Figure S2E). Simple effects analyses found increased protein expression levels of GSDMD‐N in Hypoxia group (*P* < .01), but not in HC group (*P* > .05) compared with Control group. Hypoxia + HC group had the highest expression levels of GSDMD‐N as compared with Hypoxia group (*P* < .01) and HC group (*P* < .01). Additionally, the protein expression of GSDMD‐N was significantly suppressed with NAC pretreatment (2 mmol/L) in BV‐2 microglial cells (*P* < .01) (Figure S2D, F).

### 3.12 Hypercapnia increased IL‐1β expression via inducing ROS overproduction in microglia in the hypoxic hippocampus

Significant interaction effects were observed between hypercapnia treatment and hypoxia treatment (*P* < .01) (Figure S3B). Simple effects analyses found increased protein expression levels of IL‐1β in Hypoxemia group (*P* < .01), but not in Hypercapnia group (*P* > .05) compared with Sham group. HH group showed the highest expression levels of IL‐1β in comparison with Hypoxemia group (*P* < .01) and Hypercapnia group (*P* < .01). Additionally, the protein expression of IL‐1β was significantly suppressed with NAC pretreatment (150 mg/kg) in rats (*P* < .01) (Figure S3A,C). The full Western blots of the above each group were shown in Figure S12. Double immunofluorescence was used to examine IL‐1β expression in microglia of the hippocampus (Figure S3D). Enhanced IL‐1β immunofluorescence was observed in Hypoxemia group, but not in Hypercapnia group compared with Sham group. HH group had the strongest IL‐1β fluorescence as compared with Hypoxemia group and Hypercapnia group. IL‐1β fluorescence was evidently reduced in rats given NAC pretreatment (Figure S3D).

### 3.13 15% CO_2_ increased IL‐1β expression via inducing ROS overproduction in hypoxic BV‐2 microglial cells

Significant interaction effects were observed between 0.2% O_2_ treatment and 15% CO_2_ treatment (*P* < .01) (Figure S4B). Simple effects analyses found increased protein expression levels of IL‐1β in Hypoxia group (*P* < .01), but not in HC group (*P* > .05) compared with Control group. Hypoxia + HC group had the highest expression levels of IL‐1β as compared with Hypoxia group (*P* < .01) and HC group (*P* < .01). Additionally, the protein expression of IL‐1β was significantly suppressed with NAC pretreatment (2 mmol/L) in BV‐2 microglial cells (*P* < .01) (Figure S4A, C). The full Western blots of the above each group were shown in Figure S13. Double immunofluorescence confirmed IL‐1β expression in BV‐2 microglial cells (Figure S4D). Enhanced IL‐1β immunofluorescence was observed in Hypoxia group, but not in HC group compared with Control group. Hypoxia + HC group had the strongest IL‐1β fluorescence as compared with Hypoxia group and HC group. IL‐1β fluorescence was markedly suppressed with NAC pretreatment (2 mmol/L) in BV‐2 microglial cells (Figure S4D).

### 3.14 Hypercapnia increased IL‐18 expression via inducing ROS overproduction in microglia in the hypoxic hippocampus

Significant interaction effects were observed between hypercapnia treatment and hypoxia treatment (*P* < .01) (Figure S5B). Simple effects analyses found increased protein expression levels of IL‐18 in Hypoxemia group (*P* < .01), but not in Hypercapnia group (*P* > .05) compared with Sham group. HH group showed the highest expression levels of IL‐18 in comparison with Hypoxemia group (*P* < .01) and Hypercapnia group (*P* < .01). Additionally, the protein expression of IL‐18 was significantly suppressed with NAC pretreatment (150 mg/kg) in rats (*P* < .01) (Figure S5A, C). The full Western blots of the above each group were shown in Figure S14. Double immunofluorescence was used to examine IL‐18 expression in microglia of the hippocampus (Figure S5D). Enhanced IL‐18 immunofluorescence was observed in Hypoxemia group, but not in Hypercapnia group compared with Sham group. HH group had the strongest IL‐18 fluorescence as compared with Hypoxemia group and Hypercapnia group. IL‐18 fluorescence was evidently reduced in rats given NAC pretreatment (Figure S5D).

### 3.15 15% CO_2_ increased IL‐18 expression via inducing ROS overproduction in hypoxic BV‐2 microglial cells

Significant interaction effects were observed between 0.2% O_2_ treatment and 15% CO_2_ treatment (*P* < .01) (Figure S6B). Simple effects analyses found increased protein expression levels of IL‐18 in Hypoxia group (*P* < .01), but not in HC group (*P* > .05) compared with Control group. Hypoxia + HC group had the highest expression levels of IL‐18 as compared with Hypoxia group (*P* < .01) and HC group (*P* < .01). Additionally, the protein expression of IL‐18 was significantly suppressed with NAC pretreatment (2 mmol/L) in BV‐2 microglial cells (*P* < .01) (Figure S6A, C). The full Western blots of the above each group were shown in Figure S15. Double immunofluorescence confirmed IL‐18 expression in BV‐2 microglial cells (Figure S6D). Enhanced IL‐18 immunofluorescence was observed in Hypoxia group, but not in HC group compared with Control group. Hypoxia + HC group had the strongest IL‐18 fluorescence as compared with Hypoxia group and HC group. IL‐18 fluorescence was markedly suppressed with NAC pretreatment (2 mmol/L) in BV‐2 microglial cells (Figure S6D).

## 4 DISCUSSION

The present results have shown that hypercapnia promoted pyroptosis via inhibiting mitophagy in hypoxia‐activated microglia in vitro and in vivo. This was evident by the increased CERO_2_, decreased PbtO_2_, and increased expression levels of LC3‐II, caspase‐1, GSDMD‐N, IL‐18, and IL‐1β in hypoxia‐activated microglia cells. The microglial pyroptosis in the hippocampus induced by hypoxemia/hypercapnia may perpetuate cascade inflammatory response and contribute to the neurological impairments of ARDS patients.

The present rat and cell models of hypercapnia/hypoxemia were established as described in our previous study.^9^ In Sham group, the rats were exposed to air when the ventilator settings were fixed. The PaCO_2_ levels of the rats were maintained between 35 and 45 mmHg, and the PaO_2_ levels were maintained between 90 and 100 mmHg. The normal range of human PaCO_2_ and PaO_2_ is 35‐45 and 80‐100 mmHg, respectively, which are comparable between rats and humans. In the hypercapnia/hypoxemia models, PO_2_ levels of the arterial blood and culture supernatant were maintained at 55‐60 mmHg and pH levels of the arterial blood and culture supernatant at 7.20‐7.25. These are consistent with the change of hypoxemia and permissive hypercapnia in ARDS.^27^, ^28^, ^29^, ^30^

We reported in our previous study that hypercapnia could induce IL‐1β overproduction in hypoxia‐activated microglia. However, the underlying mechanism has remained unclear. It has been reported that the expression of caspase‐1 would be upregulated after NLRP3 inflammasome being activated. Caspase‐1 could lead to IL‐18 and IL‐1β maturation and pyroptosis activation. GSDMD‐N is the executioner of pyroptosis. GSDMD‐N helped to form a pore in the plasma membrane, from that IL‐1β and IL‐18 are released.^19^, ^20^ Under hypoxic conditions, ROS is known to be critical for NLRP3 inflammasome activation.^31^, ^32^, ^33^ Furthermore, hippocampus is known to be critical for spatial and contextual memory.^34^ And the receptor of inflammatory cytokines (ie, IL‐1β)^35^ are comparatively higher in the hippocampus and are tightly linked to the deficits in hippocampus‐dependent memory.^36^ Thus, the expression levels of caspase‐1, GSDMD‐N, IL‐18, and IL‐1β in the hippocampus microglia in this study were determined, and the production of ROS was also evaluated. Significant interaction effects were observed on ROS production, caspase‐1, GSDMD‐N, IL‐18, and IL‐1β expression between hypercapnia treatment and hypoxia treatment. More importantly, we have shown that hypercapnia upregulated the expression of caspase‐1, GSDMD‐N, IL‐18, and IL‐1β in hypoxic microglia via inducing ROS overproduction. Remarkably, caspase‐1, GSDMD‐N, IL‐18, and IL‐1β expression in hypoxic microglia was downregulated when ROS was scavenged by NAC. These results indicate that hypercapnia promotes microglial pyroptosis via inducing ROS overproduction and activating the NLRP3 inflammasome.

When the levels of oxygen fall, hypoxia would damage mitochondria and induce ROS production.^22^, ^23^, ^24^ Mitophagy can eliminate damaged mitochondria, reduce ROS production, and then alleviate NLRP3 inflammasome activation.^25^ To ascertain whether hypercapnia would activate the NLRP3 inflammasome through inhibiting mitophagy in hypoxia‐activated microglia, expression of LC3‐II and p62 in the microglia was determined. p62 is a multidomain protein that interacts with phagophores through the LC3‐interacting domain and with the ubiquitinated protein aggregates through the ubiquitin‐associated domain,^37^ which promotes degradation of unwanted molecules,^38^ while p62 is an autophagy substrate and is degraded by autophagy.^39^ We found that the expression levels of LC3‐II were reduced, while p62 expression was increased by hypercapnia in the hypoxic hippocampus and BV‐2 microglia. These results indicate that hypercapnia might enhance the NLRP3 inflammasome activation in hypoxia‐activated microglial cells via inhibiting mitophagy and impairing autophagy flux.

To determine the effect of hypercapnia on oxidative metabolism of the rat brain, CERO_2_ and partial pressure of brain tissue oxygen (PbtO_2_) were examined. Hypoxemia, as a typical symptom of ARDS, decreases the oxidative metabolism rate of the brain to avoid or reduce cerebral damage via preventing the PbtO_2_ from dropping to a very low level.^40^ In this study, we showed hypercapnia alone was not enough to increase or decrease the levels of PbtO_2_ and CERO_2_. In the first 1.5 hours, the levels of PbtO_2_ were elevated by hypercapnia in the hypoxemic rats. This may be the reason why hypercapnia was neuroprotective in rats with transient global cerebral ischemia‐reperfusion injury^41^ and lateral fluid percussion injury.^42^ However, hypercapnia significantly decreased the levels of PbtO_2_ in the hypoxemic rats after 2 hours’ ventilation. It was further evidenced by the increased levels of CERO_2_ at 3 hours after ventilation. These results suggest that hypercapnia is neuroprotective within a very short period (1.5 hours), but hypercapnia could aggravate neuronal functions injury over a protracted period via increasing CERO_2_ and decreasing PbtO_2_. This is consistent with the expression of IL‐1β and IL‐18 in microglia. It was observed that hypercapnia alone may not be sufficient to increase the expression of IL‐1β and IL‐18, but hypercapnia may exert its effects in the presence of hypoxia. It was suggested that hypercapnia can induce ROS overproduction via intensifying hypoxia, may activate the NLRP3 inflammasome, and promote IL‐1β release.

In vitro, to determine the effect of a high concentration of CO_2_ on oxidative metabolism in hypoxic BV‐2 microglial cells, the oxygen consumption rate (OCR) was evaluated. There was an interaction effect on the levels of OCR between 0.2% O_2_ treatment and 15% CO_2_ treatment. The high concentration of CO_2_ treatment could increase the levels of OCR in hypoxic BV‐2 microglial cells, which were consistent with that hypercapnia increased levels of CERO_2_ in hypoxic rats. The results suggest that hypercapnia could intensify microglial hypoxia both in vivo and in vitro.

There are four limitations to this study. Firstly, it has been reported that NLRP3 inflammasome is expressed in astrocytes.^43^ However, the lack of the NLRP3 inflammasome in astrocytes being investigated in the present study is a limitation. Secondly, the present study did not prove the connection between cerebral oxygen metabolism (CERO_2_ and PbtO_2_) and mitophagy, which is an area for future research. Thirdly, the models of hypercapnia/hypoxemia were established with certain concentrations of O_2_ and CO_2_. The lack of concentration gradient of O_2_ and CO_2_ being investigated in the present study is another limitation. Fourthly, the study did not prove the upstream mechanism by which hypercapnia inhibits mitophagy. It is well documented that the PINK1‐Parkin axis functions as the core machinery for mitophagy.^44^ It was surmised that hypercapnia may exert its effect through mediating the PINK1‐Parkin signaling axis.

In summary, the present results have demonstrated the underlying mechanism whereby hypercapnia can promote pyroptosis in hypoxic microglia. In this connection, hypercapnia was found to intensify the cerebral hypoxia via increasing CERO_2_ and decreasing PbtO_2_. ROS was overproduced by hypercapnia in hypoxic microglial cells coupled with mitophagy inhibition and activation of NLRP3 inflammasome. Remarkedly, caspase‐1, GSDMD‐N, IL‐18, and IL‐1β expression was downregulated when ROS was scavenged. Thus, hypercapnia‐induced mitophagy inhibition, NLRP3 inflammasome activation, and pyroptosis in microglia may be a potential target to mitigate neuroinflammation.

## 5 CONCLUSIONS

Hypercapnia‐induced mitophagy inhibition may promote pyroptosis and enhance IL‐1β and IL‐18 release in hypoxia‐activated microglia.

## CONFLICT OF INTEREST

The authors have declared that no conflict of interest exists.

## Supporting information

Fig S1‐S15
