# Supplementary material for: Hypercapnia promotes microglial pyroptosis via inhibiting mitophagy in hypoxemic adult rats
Source: CNS Neurosci Ther. 2020 Jul 14;26(11):1134–46. doi: 10.1111/cns.13435 (PMC7564198; doi:10.1111/cns.13435)
Supplement: Supplementary file 1 — Fig S1‐S15 [file CNS-26-1134-s001.pdf]

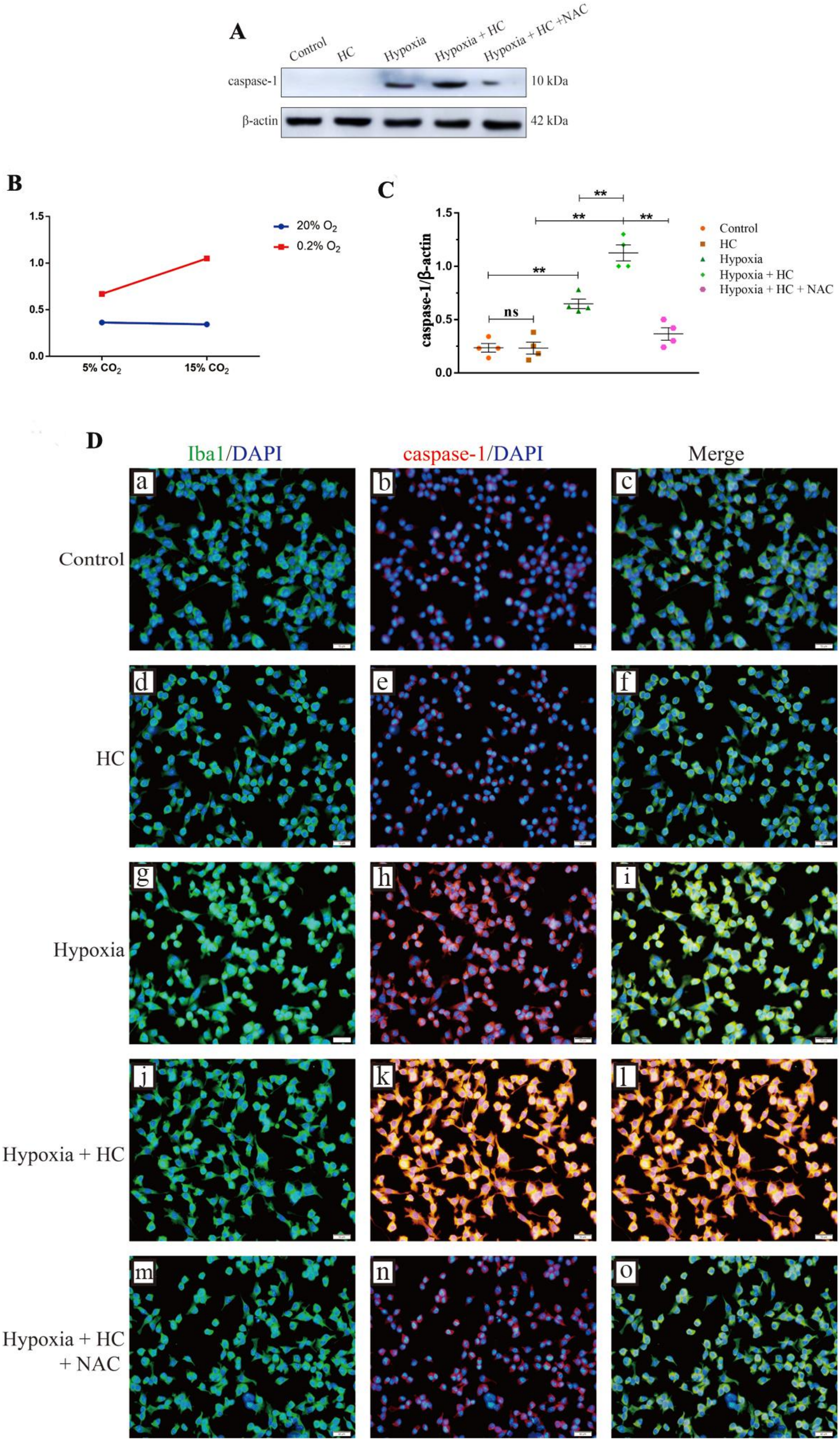
A
Control
HC
Hypoxia
Hypoxia + HC
Hypoxia + HC +NAC
caspase-1
10 kDa
β-actin
42 kDa
B
20% O2
0.2% O2
5% CO2
15% CO2
C
caspase-1/β-actin
ns
**
Control
HC
Hypoxia
Hypoxia + HC
Hypoxia + HC + NAC
D
Iba1/DAPI
caspase-1/DAPI
Merge
Control
HC
Hypoxia
Hypoxia + HC
Hypoxia + HC + NAC
a b c d e f g h i j k l m n o

Supplementary Figure 1 15% CO_2 enhanced NLRP3 inflammasome activation via inducing ROS overproduction in hypoxic BV-2 cells (n = 4).

(A) Immunoreactive bands of caspase-1 (10 kDa) and β-actin (42 kDa). (B) There is an interaction effect between 0.2% O_2 treatment and 15% CO_2 treatment ($P < 0.01$). (C) Simple effects analyses show increased protein expression levels of caspase-1 in Hypoxia group (** $P < 0.01$), but not in HC group (ns $P > 0.05$) compared with Control group. Hypoxia + HC group shows the highest expression levels of caspase-1 in comparison with Hypoxia group (** $P < 0.01$) and HC group (** $P < 0.01$). Additionally, the protein expression of caspase-1 is significantly suppressed with NAC pretreatment (2 mM) in BV-2 microglial cells (** $P < 0.01$). (D) Immunofluorescence images showing the expression of Iba1$^+$ microglia (a, d, g, j, m, green), caspase-1 (b, e, h, k, n, red), and the co-localization of caspase-1 and microglia (x, f, i, l, o). Enhanced caspase-1 immunofluorescence is evident in Hypoxia group, but not in HC group compared with Control group. Hypoxia + HC group shows the strongest caspase-1 fluorescence as compared with Hypoxia group and HC group. Caspase-1 fluorescence is drastically reduced with NAC pretreatment (2 mM) in BV-2 microglial cells. Scale bars: 10 μm. ns, non-significant; HC group, high concentration of carbon dioxide group.

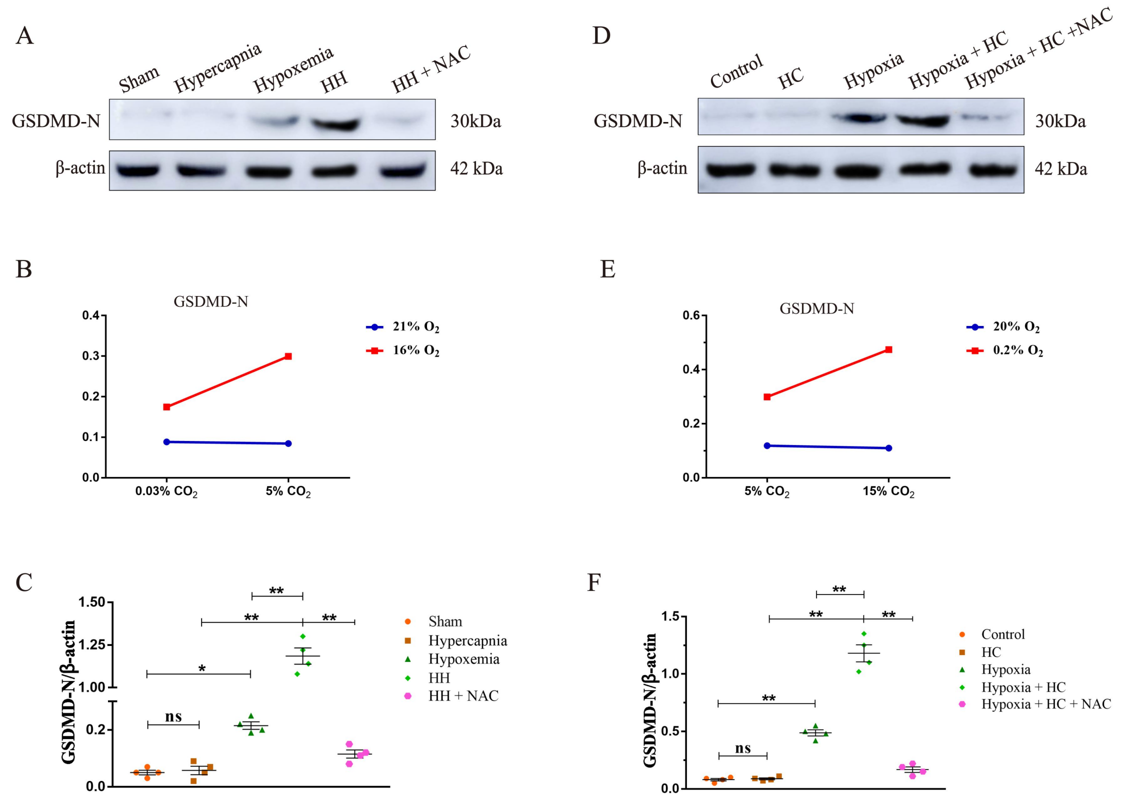

Supplementary Figure 2 Hypercapnia increased GSDMD-N expression via inducing ROS overproduction in microglia *in vivo* and *in vitro* (n = 4).

(A, D) Immunoreactive bands of GSDMD-N (30 kDa) and β-actin (42 kDa). (B) There is an interaction effect between hypercapnia treatment and hypoxia treatment ($P < 0.01$). (E) There is an interaction effect between 0.2% O_2 treatment and 15% CO_2 treatment (** $P < 0.01$). (C) Simple effects analyses show increased protein expression levels of GSDMD-N in Hypoxemia group (* $P < 0.05$), but not in Hypercapnia group (ns $P > 0.05$) compared with Sham group. HH group shows the highest expression levels of GSDMD-N in comparison with Hypoxemia group (** $P < 0.01$) and Hypercapnia group (** $P < 0.01$). Additionally, the protein expression of GSDMD-N is significantly suppressed with NAC pretreatment in rats (** $P < 0.01$). (F) Simple effects analyses show increased protein expression levels of GSDMD-N in Hypoxia group (** $P < 0.01$), but not in HC group (ns $P > 0.05$) compared with Control group. Hypoxia + HC group has the highest expression levels of GSDMD-N as compared with Hypoxia group (** $P < 0.01$) and HC group (** $P < 0.01$). Additionally, the protein expression of GSDMD-N is significantly suppressed with NAC pretreatment in BV-2 microglial cells (** $P < 0.01$). GSDMD-N, gasdermin D-N domains. ns, non-significant; Sham group, sham-operated group; HH group,

hypercapnia + hypoxemia group; HC group, high concentration of carbon dioxide group. The concentrations of O_2 and CO_2 in the air are 21% and 0.03%.

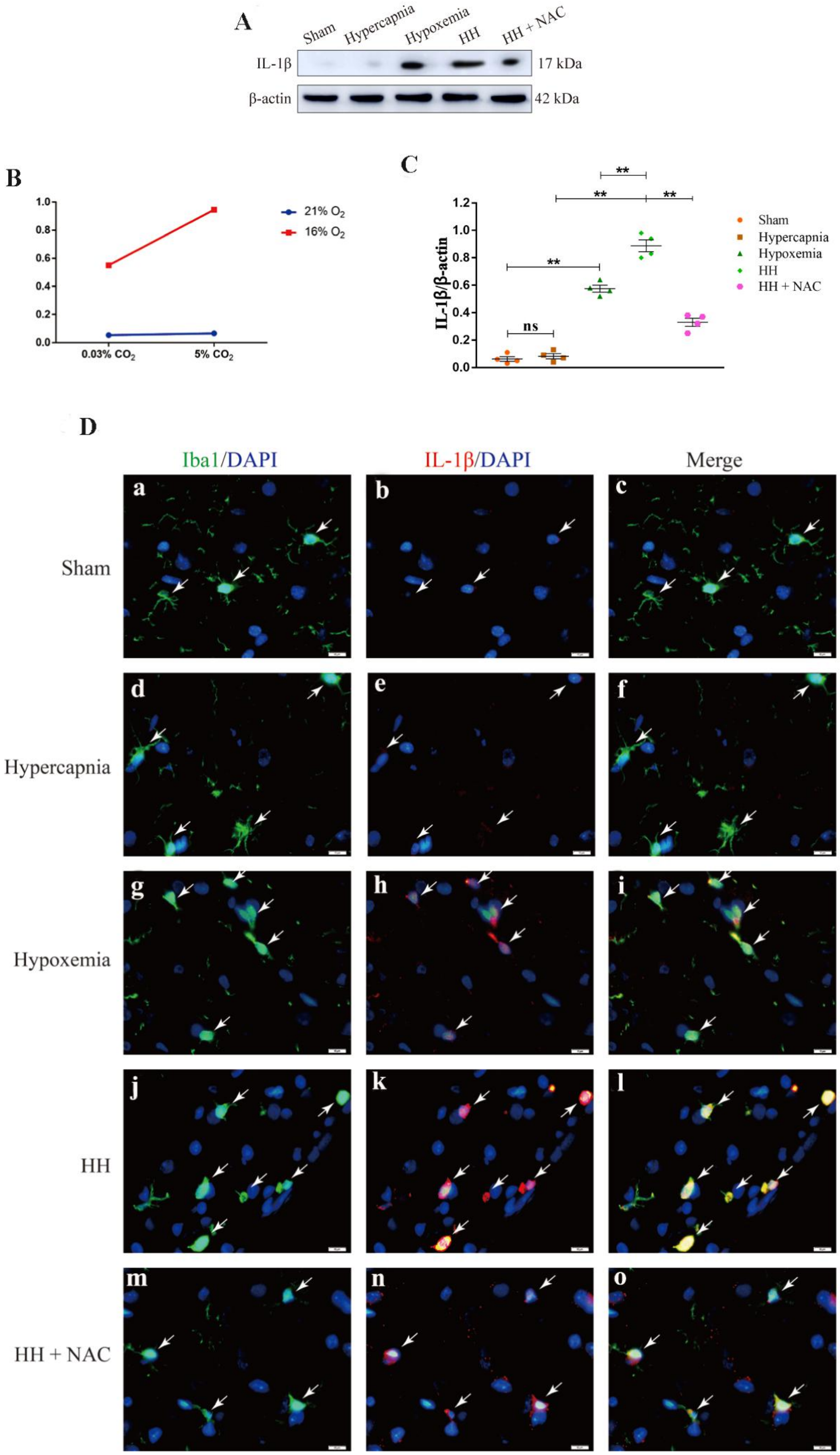
A
Sham
Hypercapnia
Hypoxemia
HH
HH + NAC
IL-1β
17 kDa
β-actin
42 kDa
B
1.0
0.8
0.6
0.4
0.2
0.0
0.03% CO2
5% CO2
21% O2
16% O2
C
IL-1β/β-actin
1.2
1.0
0.8
0.6
0.4
0.2
0.0
**
ns
Sham
Hypercapnia
Hypoxemia
HH
HH + NAC
D
Iba1/DAPI
IL-1β/DAPI
Merge
a
b
c
Sham
d
e
f
Hypercapnia
g
h
i
Hypoxemia
j
k
l
HH
m
n
o
HH + NAC

Supplementary Figure 3 Hypercapnia increased IL-1β expression via inducing ROS overproduction in microglia of hypoxic hippocampus (n = 4).

(A) Immunoreactive bands of IL-1β (17 kDa) and β-actin (42 kDa). (B) There is an interaction effect between hypoxia treatment and hypercapnia treatment ($P < 0.01$). (C) Simple effects analyses show increased protein expression levels of IL-1β in Hypoxemia group (** $P < 0.01$), but not in Hypercapnia group (ns $P > 0.05$) compared with Sham group. HH group has the highest expression levels of IL-1β as compared with Hypoxemia group (** $P < 0.01$) and Hypercapnia group (** $P < 0.01$). The protein expression of IL-1β is significantly suppressed with NAC pretreatment in rats (** $P < 0.01$). (D) Immunofluorescence images showing the expression of Iba1$^+$ microglia (a, d, g, j, m, green), IL-1β (b, e, h, k, n, red), and the co-localization of IL-1β and microglia (x, f, i, l, o). Enhanced IL-1β immunofluorescence is evident in Hypoxemia group, but not in Hypercapnia group compared with Sham group. HH group has the strongest IL-1β fluorescence as compared with Hypoxemia group and Hypercapnia group. IL-1β fluorescence is markedly reduced in rats given NAC pretreatment. Scale bars: 10 μm. IL-1β, interleukin-1 beta; ns, non-significant; Sham group, sham-operated group; HH group, hypercapnia + hypoxemia group. The concentrations of O_2 and CO_2 in the air are 21% and 0.03%.

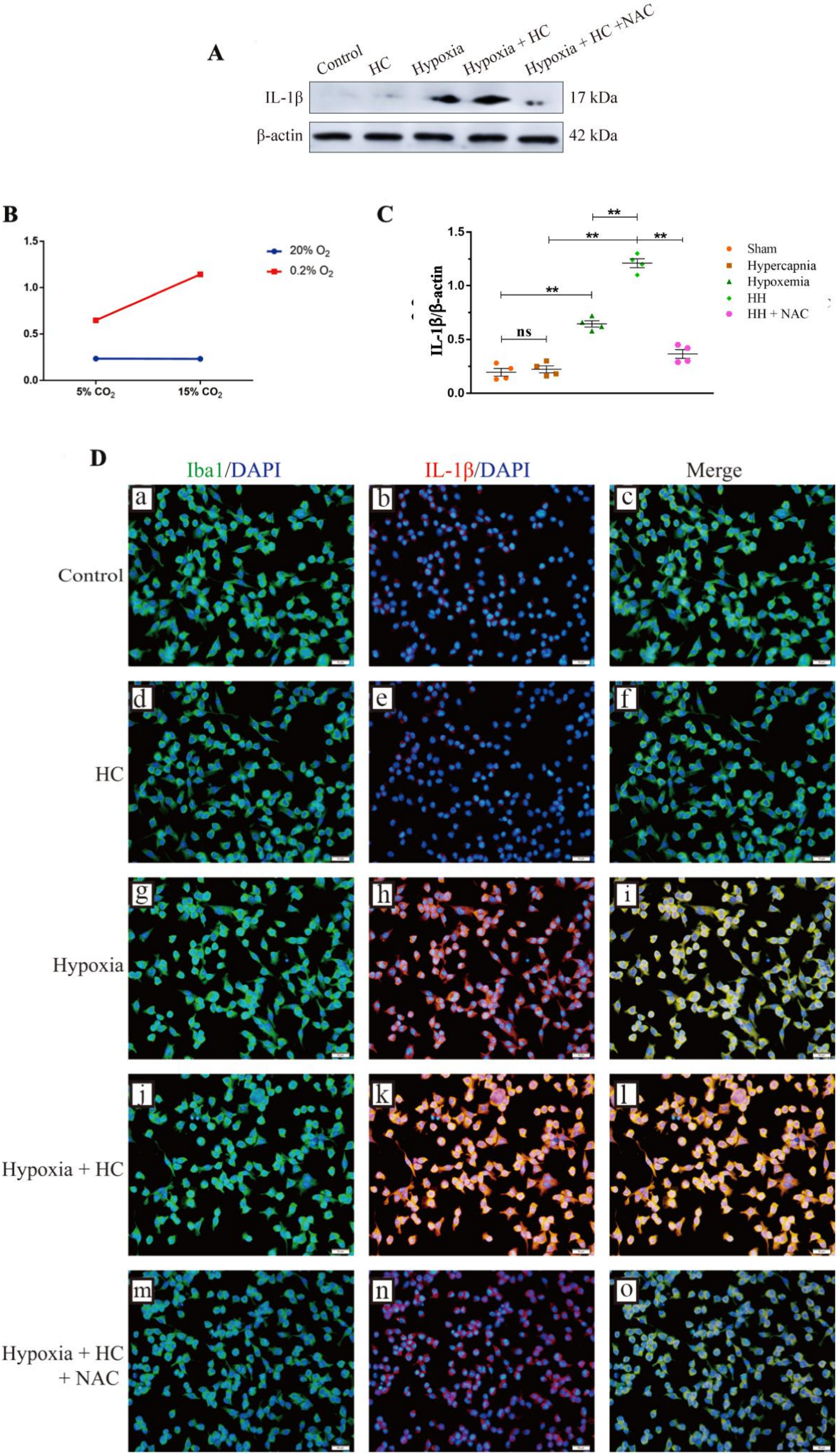

A
Control
HC
Hypoxia
Hypoxia + HC
Hypoxia + HC +NAC
IL-1β
17 kDa
β-actin
42 kDa
B
1.5
1.0
0.5
0.0
5% CO2
15% CO2
20% O2
0.2% O2
C
IL-1β/β-actin
1.5
1.0
0.5
0.0
ns
**
Sham
Hypercapnia
Hypoxemia
HH
HH + NAC
D
Iba1/DAPI
IL-1β/DAPI
Merge
Control
HC
Hypoxia
Hypoxia + HC
Hypoxia + HC + NAC
a
b
c
d
e
f
g
h
i
j
k
l
m
n
o

Supplementary Figure 4 15% CO_2 increased IL-1β expression via inducing ROS overproduction in hypoxic BV-2 cells (n = 4).

(A) Immunoreactive bands of IL-1β (17 kDa) and β-actin (42 kDa). (B) There is an interaction effect between 0.2% O_2 treatment and 15% CO_2 treatment ($P < 0.01$). (C) Simple effects analyses show increased protein expression levels of IL-1β in Hypoxia group (** $P < 0.01$), but not in HC group (ns $P > 0.05$) compared with Control group. Hypoxia + HC group shows the highest expression levels of IL-1β as compared with Hypoxia group (** $P < 0.01$) and HC group (** $P < 0.01$). Additionally, the protein expression of IL-1β is significantly suppressed with NAC pretreatment in BV-2 microglial cells (** $P < 0.01$). (D) Immunofluorescence images showing Iba1$^+$ microglia (a, d, g, j, m, green), IL-1β labeling (b, e, h, k, n, red), and their co-localization (x, f, i, l, o). Enhanced IL-1β immunofluorescence is evident in Hypoxia group, but not in HC group compared with Control group. Hypoxia + HC group displays the strongest IL-1β fluorescence when compared with Hypoxia group and HC group. IL-1β fluorescence is markedly reduced with NAC pretreatment (2 mM) in BV-2 microglial cells. Scale bars: 10 μm. IL-1β, interleukin-1 beta; ns, non-significant; HC group, high concentration of carbon dioxide group.

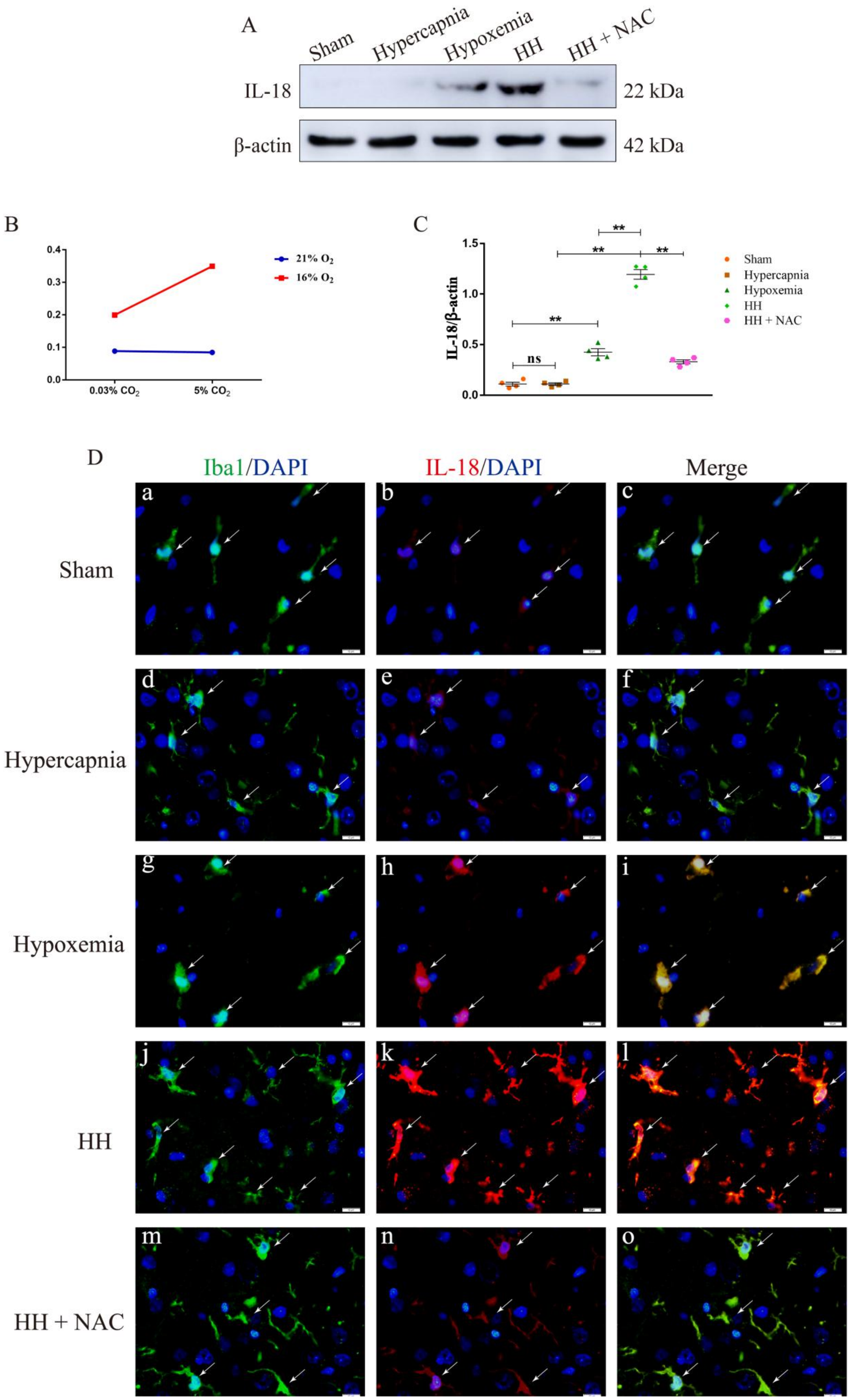
A
Sham
Hypercapnia
Hypoxemia
HH
HH + NAC
IL-18
22 kDa
β-actin
42 kDa
B
0.03% CO2
5% CO2
21% O2
16% O2
C
IL-18/β-actin
ns
**
Sham
Hypercapnia
Hypoxemia
HH
HH + NAC
D
Iba1/DAPI
IL-18/DAPI
Merge
Sham
Hypercapnia
Hypoxemia
HH
HH + NAC

Supplementary Figure 5 Hypercapnia increased IL-18 expression via inducing ROS overproduction in microglia of hypoxic hippocampus (n = 4).

(A) Immunoreactive bands of IL-18 (22 kDa) and β-actin (42 kDa). (B) There is an interaction effect between hypoxia treatment and hypercapnia treatment ($P < 0.01$). (C) Simple effects analyses show increased protein expression levels of IL-18 in Hypoxemia group (** $P < 0.01$), but not in Hypercapnia group (ns $P > 0.05$) compared with Sham group. HH group has the highest expression levels of IL-18 as compared with Hypoxemia group (** $P < 0.01$) and Hypercapnia group (** $P < 0.01$). The protein expression of IL-18 is significantly suppressed with NAC pretreatment in rats (** $P < 0.01$). (D) Immunofluorescence images showing the expression of Iba1$^+$ microglia (a, d, g, j, m, green), IL-18 (b, e, h, k, n, red), and the co-localization of IL-18 and microglia (x, f, i, l, o). Enhanced IL-18 immunofluorescence is evident in Hypoxemia group, but not in Hypercapnia group compared with Sham group. HH group has the strongest IL-18 fluorescence as compared with Hypoxemia group and Hypercapnia group. IL-18 fluorescence is markedly reduced in rats given NAC pretreatment. Scale bars: 10 μm. IL-18, interleukin-18; ns, non-significant; Sham group, sham-operated group; HH group, hypercapnia + hypoxemia group. The concentrations of O_2 and CO_2 in the air are 21% and 0.03%.

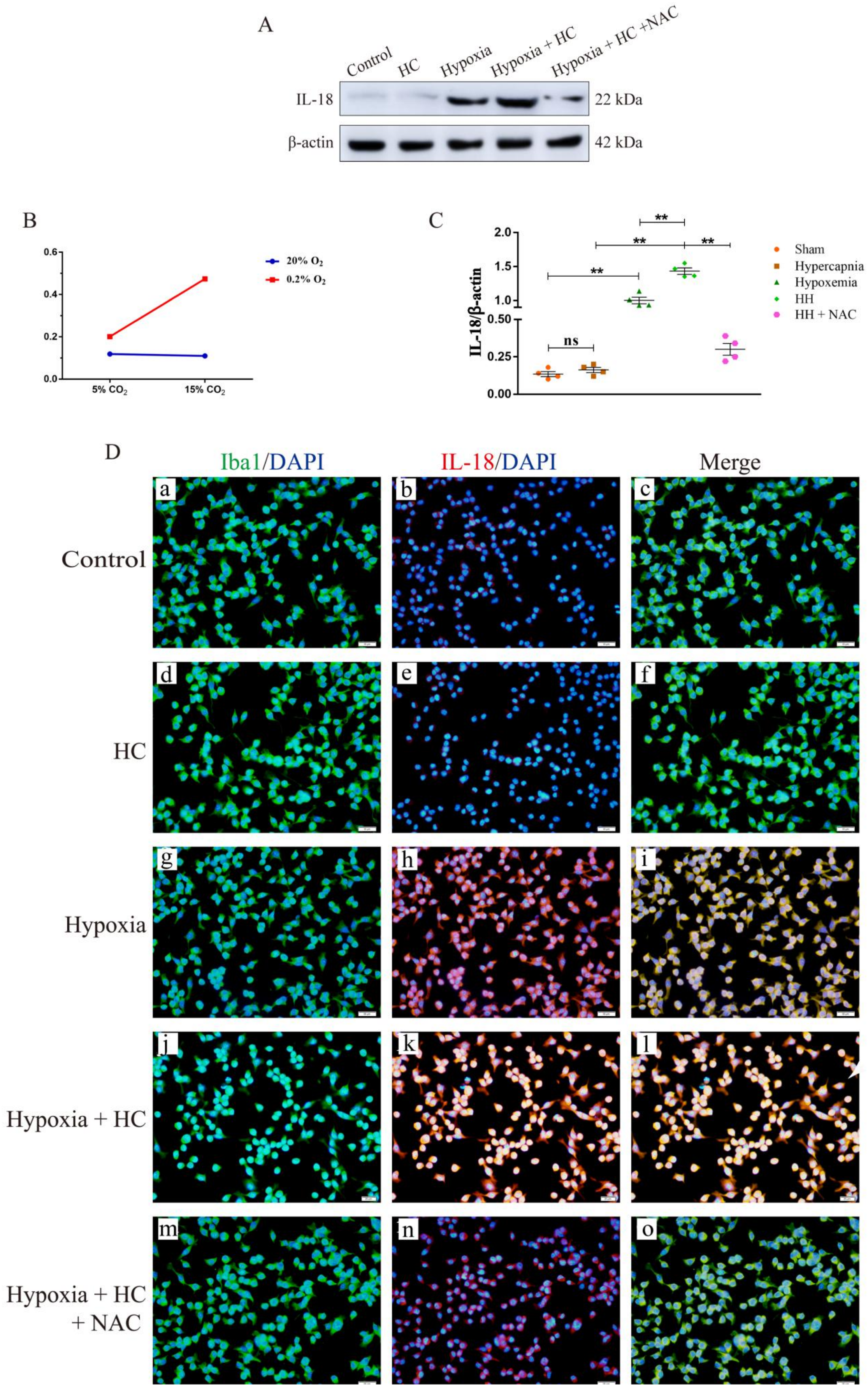
A
Control
HC
Hypoxia
Hypoxia + HC
Hypoxia + HC +NAC
IL-18
22 kDa
β-actin
42 kDa
B
0.6
0.4
0.2
0.0
5% CO2
15% CO2
20% O2
0.2% O2
C
IL-18/β-actin
2.0
1.5
1.0
0.50
0.25
0.00
ns
**
Sham
Hypercapnia
Hypoxemia
HH
HH + NAC
D
Iba1/DAPI
IL-18/DAPI
Merge
Control
HC
Hypoxia
Hypoxia + HC
Hypoxia + HC
+ NAC
a
b
c
d
e
f
g
h
i
j
k
l
m
n
o

Supplementary Figure 6 15% CO_2 increased IL-18 expression via inducing ROS overproduction in hypoxic BV-2 cells (n = 4).

(A) Immunoreactive bands of IL-18 (17 kDa) and β-actin (42 kDa). (B) There is an interaction effect between 0.2% O_2 treatment and 15% CO_2 treatment ($P < 0.01$). (C) Simple effects analyses show increased protein expression levels of IL-18 in Hypoxia group (** $P < 0.01$), but not in HC group (ns $P > 0.05$) compared with Control group. Hypoxia + HC group shows the highest expression levels of IL-18 as compared with Hypoxia group (** $P < 0.01$) and HC group (** $P < 0.01$). Additionally, the protein expression of IL-18 is significantly suppressed with NAC pretreatment in BV-2 microglial cells (** $P < 0.01$). (D) Immunofluorescence images showing Iba1$^+$ microglia (a, d, g, j, m, green), IL-18 labeling (b, e, h, k, n, red), and their co-localization (x, f, i, l, o). Enhanced IL-18 immunofluorescence is evident in Hypoxia group, but not in HC group compared with Control group. Hypoxia + HC group displays the strongest IL-18 fluorescence when compared with Hypoxia group and HC group. IL-18 fluorescence is markedly reduced with NAC pretreatment (2 mM) in BV-2 microglial cells. Scale bars: 10 μm. IL-18, interleukin-18; ns, non-significant; HC group, high concentration of carbon dioxide group.

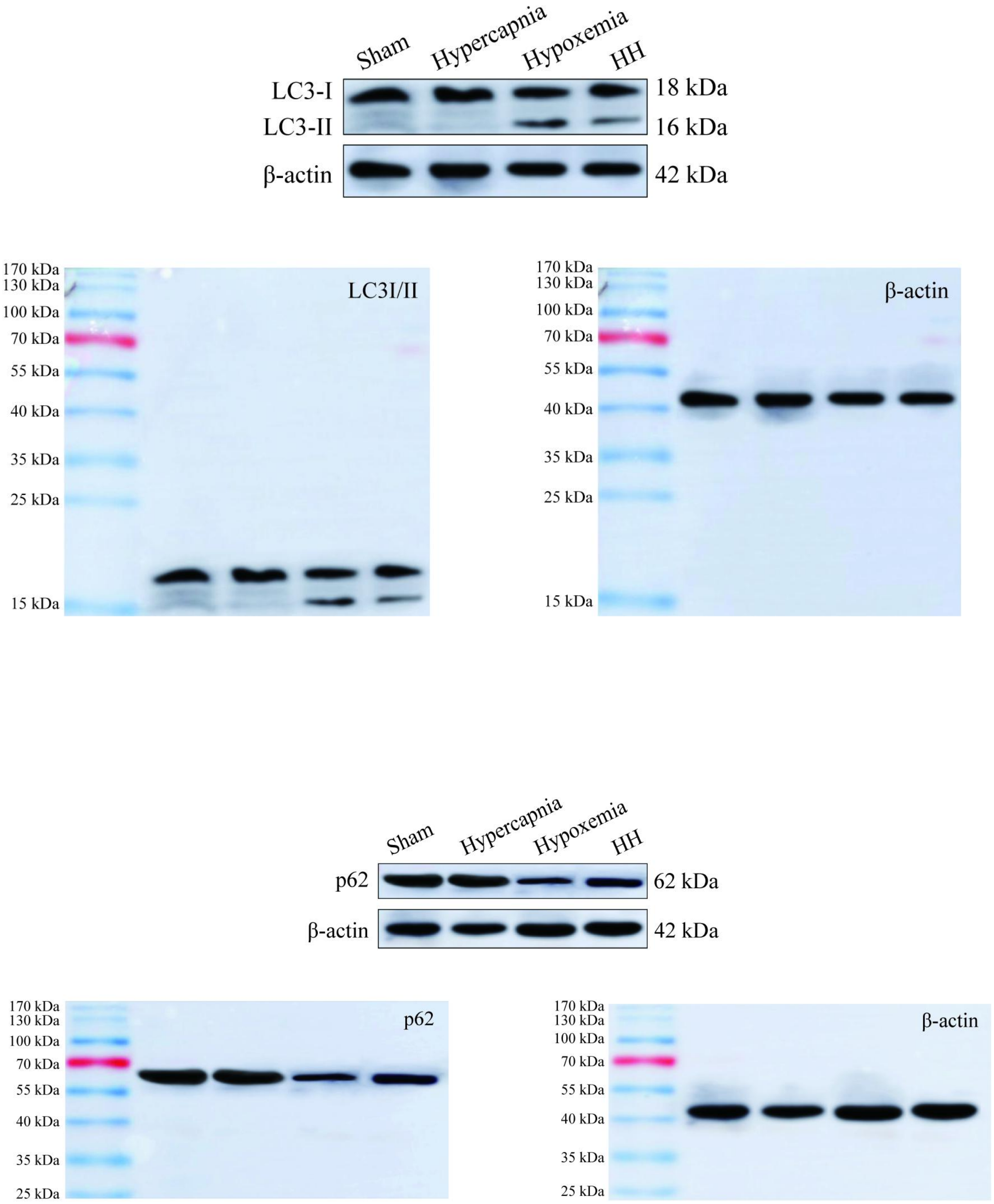

Supplementary Figure 7 Full unedited blots for Figure 3.

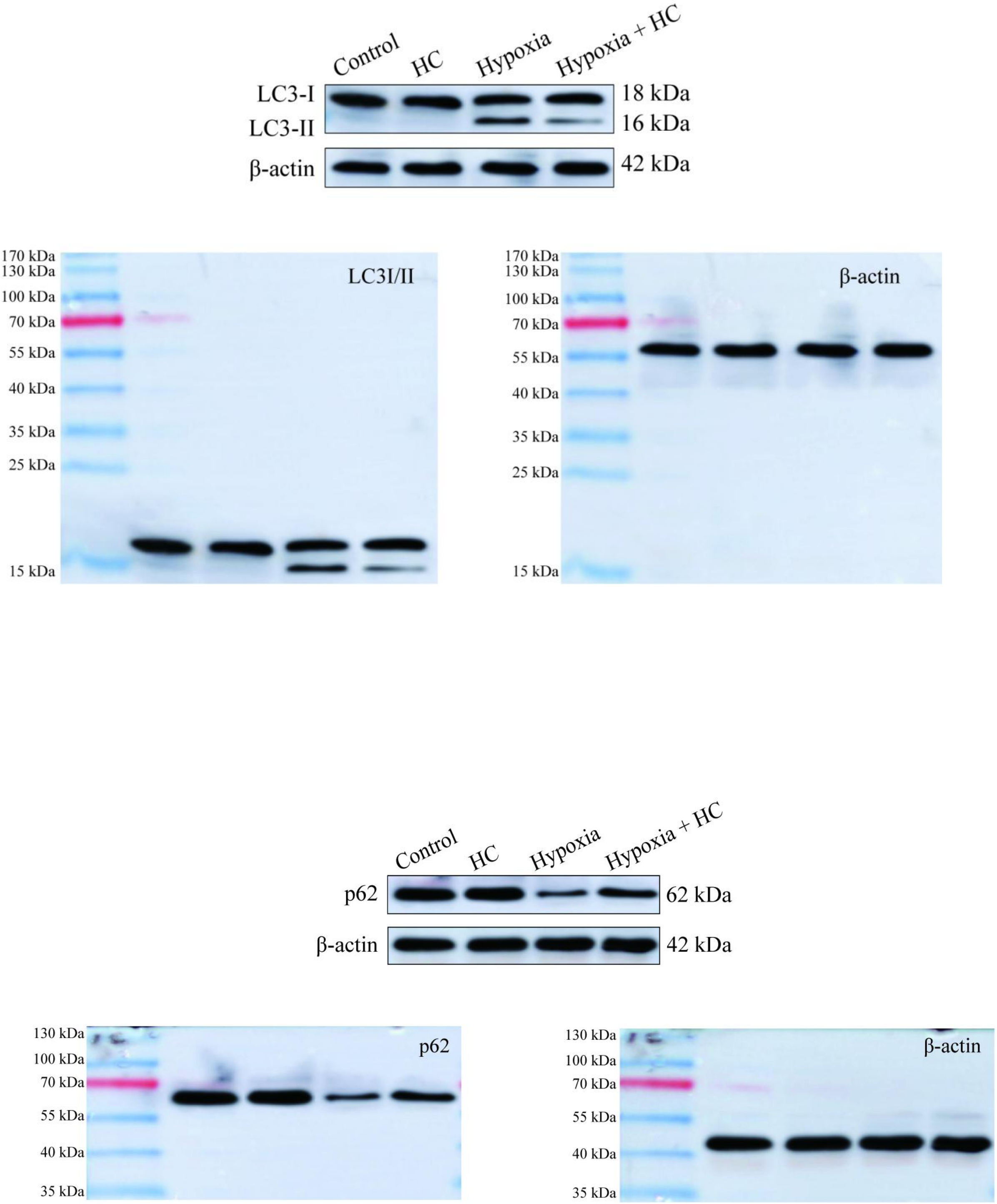

Supplementary Figure 8 Full unedited blots for Figure 4.

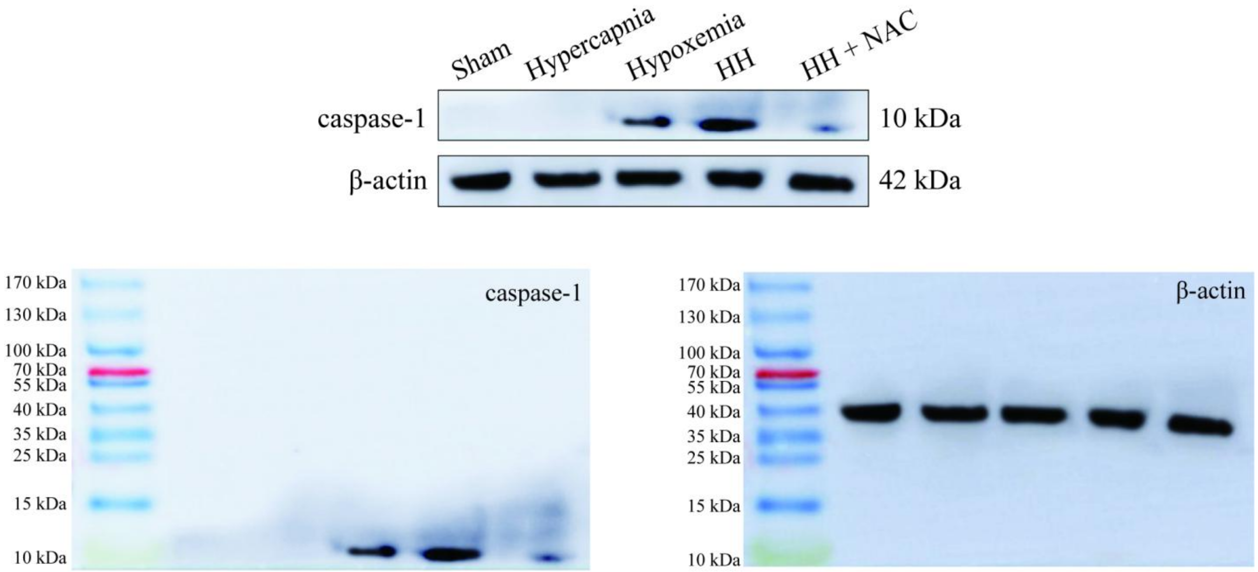

Supplementary Figure 9 Full unedited blots for Figure 6.

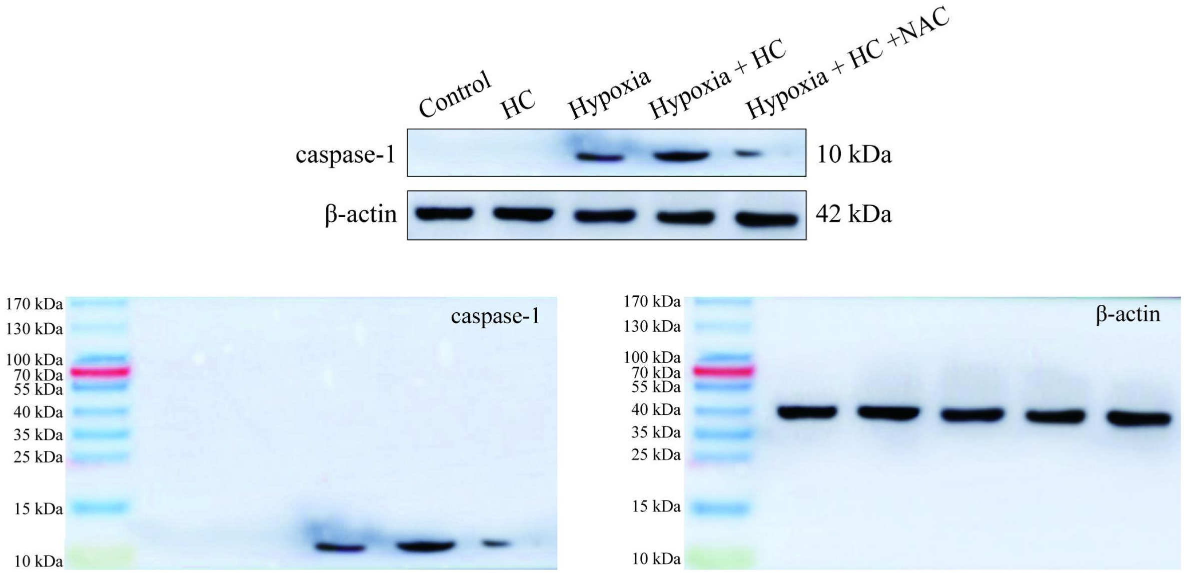

Supplementary Figure 10 Full unedited blots for Supplementary Figure 1.

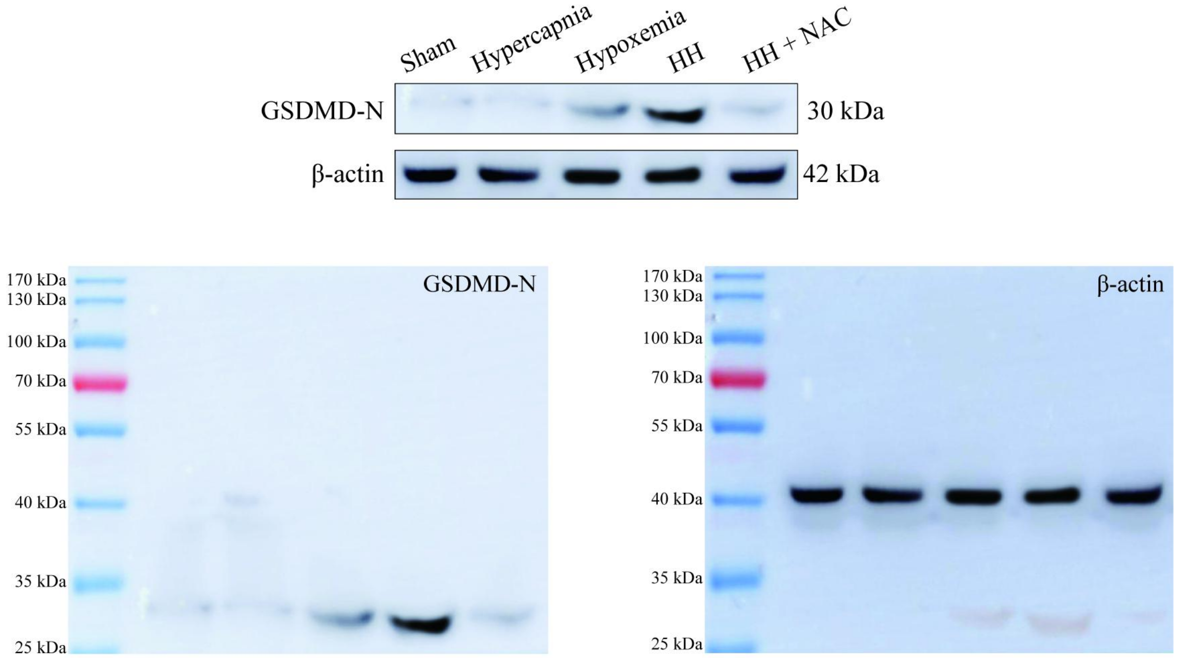

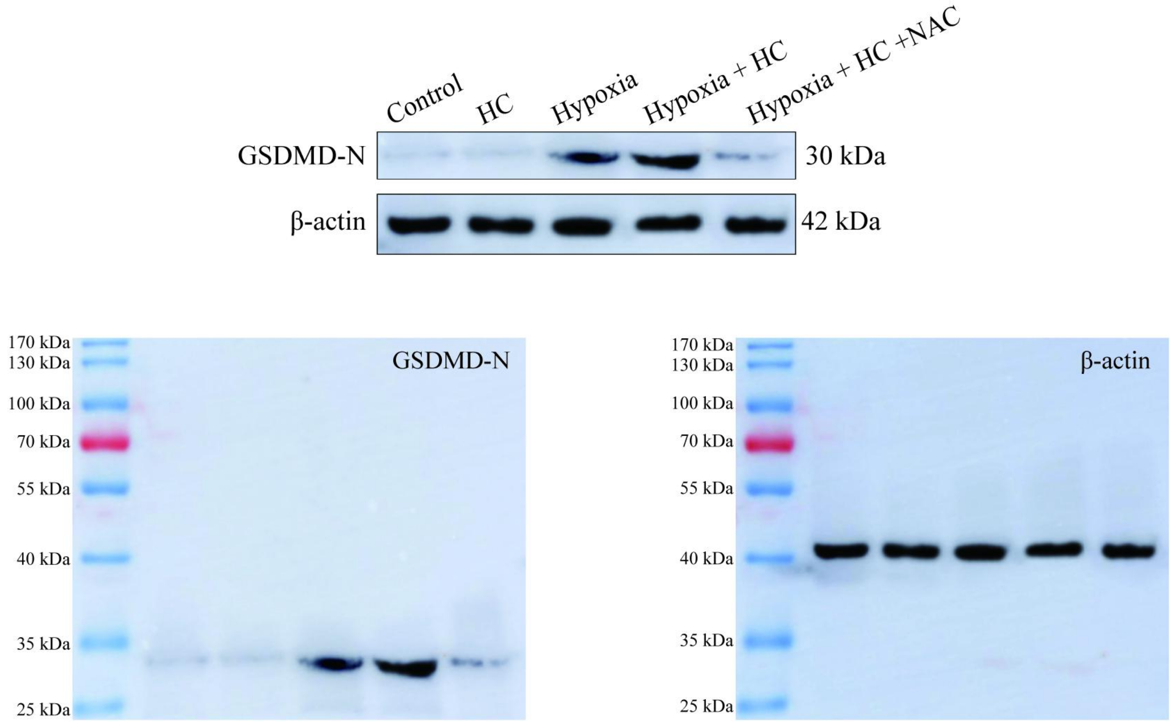

Supplementary Figure 11 Full unedited blots for Supplementary Figure 2.

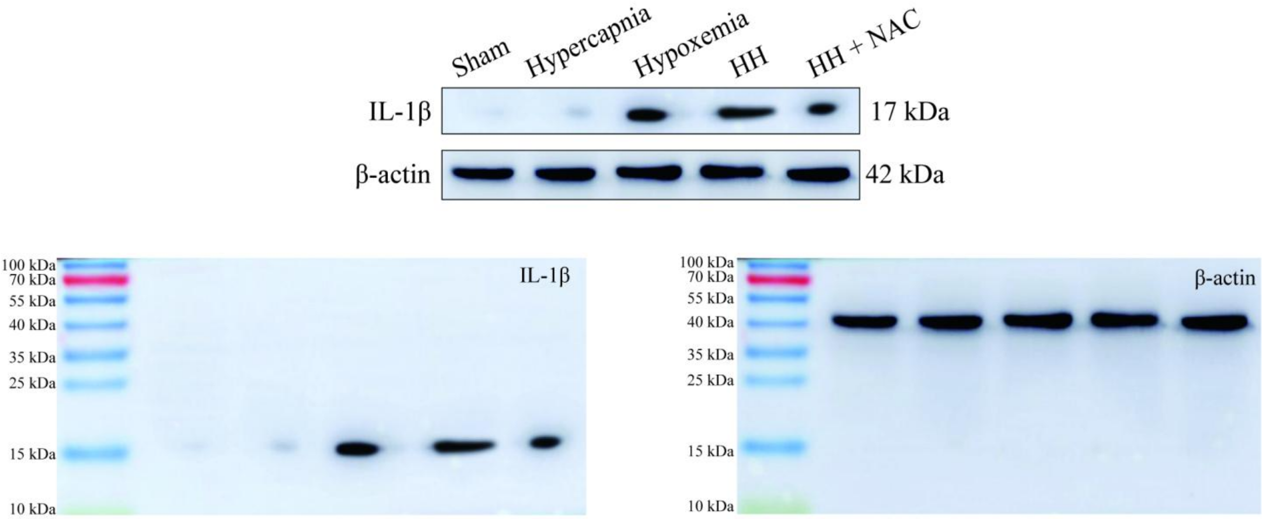

Supplementary Figure 12 Full unedited blots for Supplementary Figure 3.

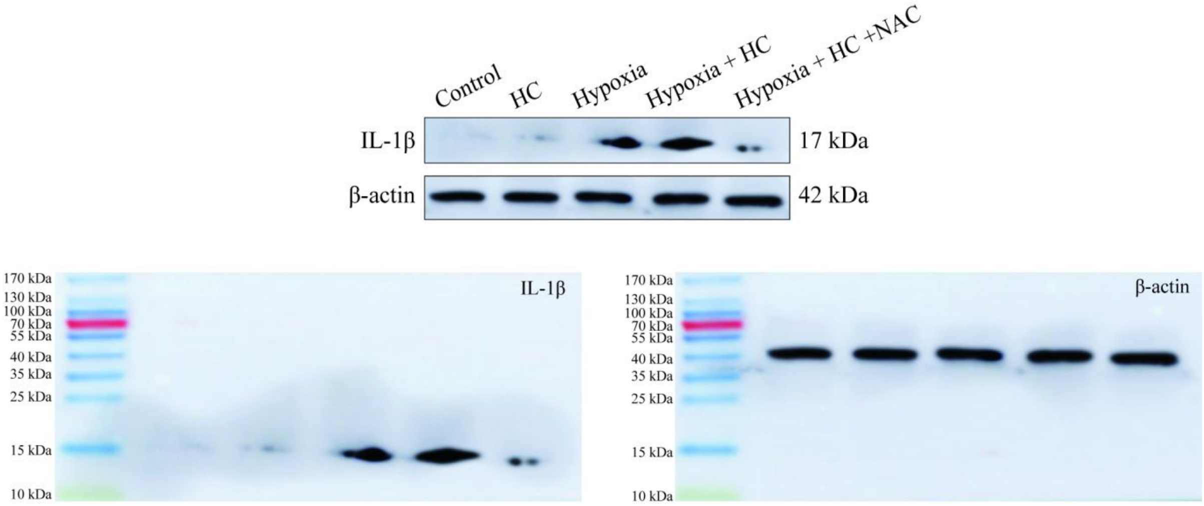

Supplementary Figure 13 Full unedited blots for Supplementary Figure 4.

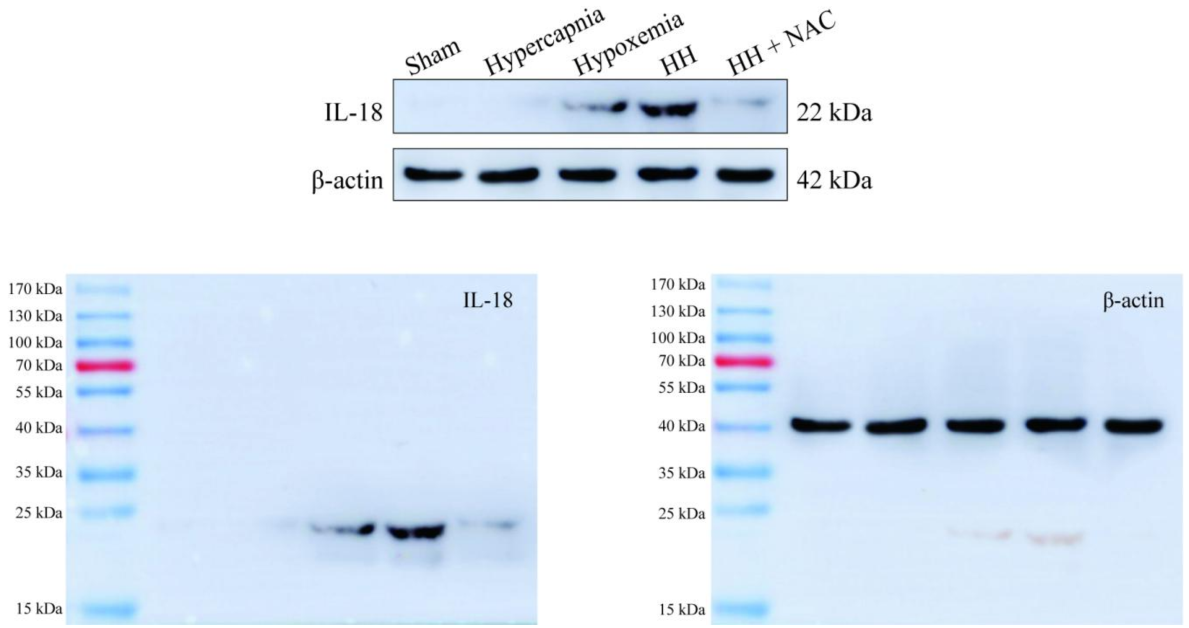

Supplementary Figure 14 Full unedited blots for Supplementary Figure 5.

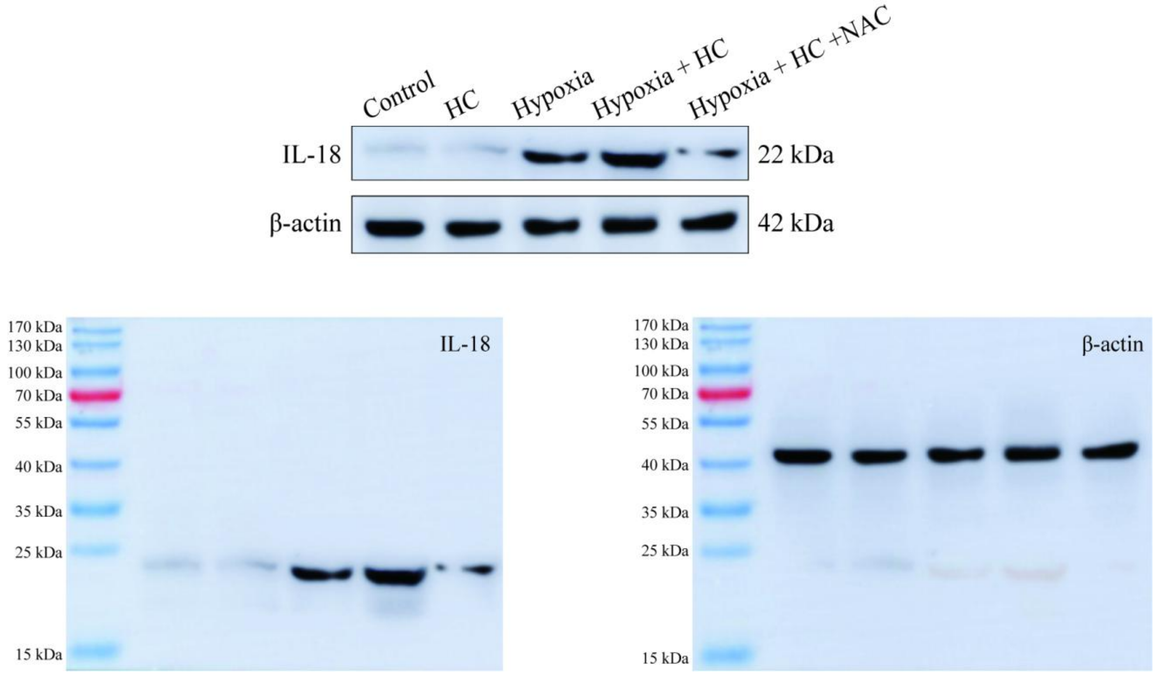

Supplementary Figure 15 Full unedited blots for Supplementary Figure 6.
